# Functional DNA-based cytoskeletons for synthetic cells

**DOI:** 10.1038/s41557-022-00945-w

**Published:** 2022-06-20

**Authors:** Pengfei Zhan, Kevin Jahnke, Na Liu, Kerstin Göpfrich

**Affiliations:** 1 2nd Physics Institute, University of Stuttgart, Stuttgart, Germany; 2 Max Planck Institute for Solid State Research, Stuttgart, Germany; 3 Biophysical Engineering Group, Max Planck Institute for Medical Research, Heidelberg, Germany; 4 Department of Physics and Astronomy, Heidelberg University, Heidelberg, Germany

**Keywords:** Biopolymers, DNA and RNA, Synthetic biology, DNA nanostructures, Permeation and transport

## Abstract

The cytoskeleton is an essential component of a cell. It controls the cell shape, establishes the internal organization, and performs vital biological functions. Building synthetic cytoskeletons that mimic key features of their natural counterparts delineates a crucial step towards synthetic cells assembled from the bottom up. To this end, DNA nanotechnology represents one of the most promising routes, given the inherent sequence specificity, addressability and programmability of DNA. Here we demonstrate functional DNA-based cytoskeletons operating in microfluidic cell-sized compartments. The synthetic cytoskeletons consist of DNA tiles self-assembled into filament networks. These filaments can be rationally designed and controlled to imitate features of natural cytoskeletons, including reversible assembly and ATP-triggered polymerization, and we also explore their potential for guided vesicle transport in cell-sized confinement. Also, they possess engineerable characteristics, including assembly and disassembly powered by DNA hybridization or aptamer–target interactions and autonomous transport of gold nanoparticles. This work underpins DNA nanotechnology as a key player in building synthetic cells.

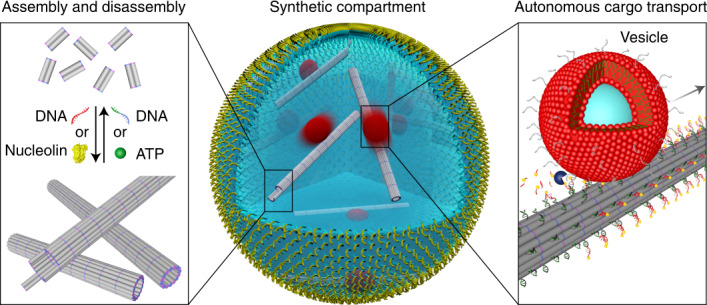

## Abstract (web summary)

Cytoskeletons are essential components of cells that perform a variety of tasks, and artificial cytoskeletons that perform these functions are required for the bottom-up assembly of synthetic cells. Now, a multi-functional cytoskeleton mimic has been engineered from DNA, consisting of confined DNA filaments that are capable of reversible self-assembly and transport of gold nanoparticles and vesicular cargo.

## Main

The cytoskeleton in a living cell functions far more powerfully than is suggested by the etymon ‘skeleton’. As well as serving as a mechanical support, it is involved in diverse cellular processes, ranging from cell division and motility to signal transduction and intracellular transport^1,2^. The multi-functional nature of the cytoskeleton means that there are great challenges in building biomimetic analogues in pursuit of bottom-up cell-free synthetic cells. Meanwhile, in the field of DNA nanotechnology, a variety of DNA-based multi-functional devices have been accomplished beyond nanoscopic art and sophisticated nanoarchitectures^2–4^. The remarkable examples achieved so far include a plethora of biomimetic systems, such as DNA-based ion channels^5,6^, walkers^7,8^, rotors^9,10^ and assembly lines^11^, which closely resemble the molecular machines in living cells. Recently, preliminary attempts have been made to achieve the stimuli-responsive assembly of DNA-based filaments^12–15^ and recently also reconstituted into cell-sized confinement^16^. However, there is still a lack of cytoskeleton mimics with sufficiently controlled multi-functionality in cell-sized compartments to master the required complexity and advance a crucial step towards synthetic cells. In this Article we demonstrate DNA-based cytoskeleton mimics that possess the most representative characteristics of natural cytoskeletons, including compartmentalization, adenosine triphosphate (ATP)-triggered polymerization and reversible assembly, as well as data that suggest intracellular cargo transport. We also show that these DNA-based cytoskeleton mimics can be programmably designed to assemble and disassemble, powered by DNA hybridization or aptamer–target interactions with unprecedented degrees of freedom.

## Results

### Design of the DNA cytoskeletons

**Fig. 1:**
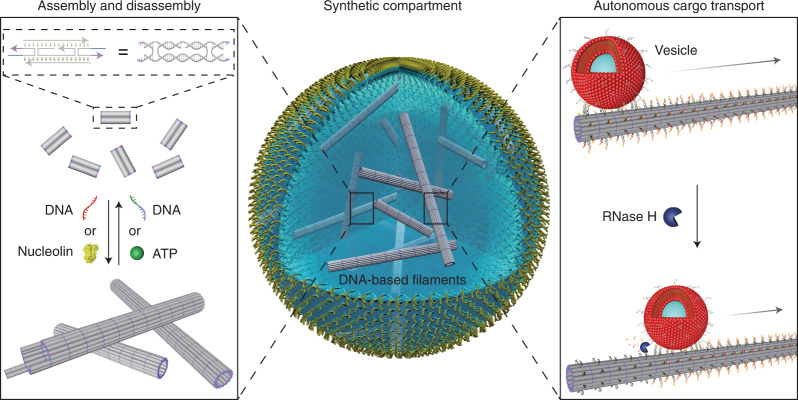
Functional DNA-based cytoskeletons for synthetic cells. Schematic of a cell-sized microfluidic droplet, containing multi-functional DNA-based filaments. The DNA-based filaments undergo dynamic assembly and disassembly triggered by strand-displacement reactions or aptamer–target interactions. Moreover, apparent guided directional transport of organic lipid membrane vesicles or inorganic gold nanoparticles along the filament tracks is powered by ribonuclease H (RNase H)-mediated hydrolysis.

**Fig. 2:**
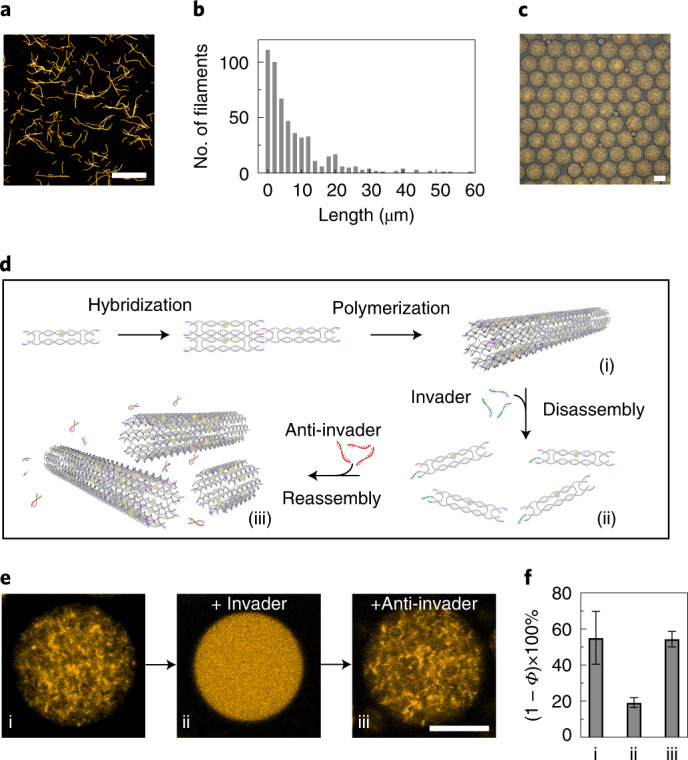
Assembly and disassembly of the DNA-based filaments in cell-sized confinement. **a**, Confocal microscopy image of the Cy3-labelled DNA-based filaments (excitation wavelength, *λ*_ex_ = 561 nm). Scale bar, 20 μm. **b**, Histogram of the filament lengths determined by confocal microscopy, showing a mean length of 7.74 μm (*n* = 516). **c**, Overlay of the confocal and bright-field overview images of the monodisperse microfluidic water-in-oil droplets containing Cy3-labelled DNA-based filaments (excitation wavelength, *λ*_ex_ = 561 nm). Scale bar, 50 μm. **d**, Schematic of the DNA tile design with toeholds^13^. Addition of the invader strands leads to the disassembly of the filaments, whereas addition of the anti-invader strands leads to reassembly. **e**, Representative confocal images of the DNA-based filaments encapsulated into droplets (i) before and (ii) after the addition of the invader strands and (iii) after the addition of the anti-invader strands. Upon addition of the invader strands the filaments are disassembled, leading to a homogeneous distribution of the fluorescence signals inside the droplet. Scale bar, 20 μm. **f**, Histogram of the porosity ((1 − *Φ*) × 100%, reflecting the degree of polymerization) of the DNA-based filaments encapsulated into water-in-oil droplets (i) in the absence, (ii) in the presence of the invader strands (10 μM) and (iii) after addition of the anti-invader strands (37.5 μM). Error bars correspond to the standard deviation of *n* ≥ 5 droplets. Source data

Figure 1 presents a conceptualized illustration of our synthetic system, including compartmentalization of different functional components in cell-sized confinement using microfluidic technologies, assembly and disassembly of DNA-based filaments triggered by DNA hybridization or aptamer–target interactions, as well as apparent autonomous transport of lipid membrane vesicles or gold nanoparticles along the filaments, powered by ribonuclease H (RNase H)-mediated hydrolysis. To implement DNA-based filaments as cytoskeleton mimics, a DNA tile design containing five individual DNA strands^12,17^ was employed to yield micrometre-long hollow DNA tubes through self-assembly. As shown in Fig. 1, the DNA tile is functionalized with four short sticky ends (light purple and blue) that serve as binding domains (Supplementary Fig. 1 and Supplementary Table 1 provide a complete list of DNA sequences). Such an arrangement of the sticky ends can guide the interaction of the DNA tiles to form tubular DNA filaments with a range of circumferences that comprise six to eight tiles^13,17,18^. The confocal microscopy image of the formed structures in Fig. 2a confirms the successful assembly of these filaments. Structural analysis using atomic force microscopy (AFM) images reveals an average filament diameter of 12 nm (Supplementary Fig. 2), corresponding to six tiles (12 DNA duplexes) around the tubular cross-sections. The assembly conditions, including the DNA tile concentration, buffer conditions and temperature, have been optimized to provide a high yield of correctly assembled long filaments (Methods and Supplementary Figs. 2–4). Confocal microscopy analysis revealed an average length of 7.74 μm (Fig. 2b and Supplementary Fig. 5). All the components for filament assembly were then encapsulated into cell-sized droplets using microfluidics (Supplementary Fig. 6). The confocal microscopy image in Fig. 2c shows the formation of monodisperse droplets that confine meshworks of the DNA-based filaments inside their lumina. The average droplet diameter is engineerable and can be varied to meet different experimental requirements. As shown in Supplementary Video 1, the filaments remain dynamic, displaying constant remodelling and rearrangement of the assemblies in three-dimensional (3D) confinement.

### Reversible assembly by strand displacement

Capitalizing on the unique programmability of DNA, dynamic assembly and disassembly of the filaments is enabled by sequential toehold-mediated DNA-strand-displacement reactions^19^. More specifically, as shown in Fig. 2d, the DNA tiles^20^ are modified with toeholds that can be displaced following the addition of the invader strands before encapsulation^13^ (Supplementary Fig. 7). This results in the disassembly of the filaments, giving rise to a homogeneous distribution of the fluorophore-tagged DNA inside the droplet lumen (Fig. 2e). Subsequent addition of the anti-invader strands directly before encapsulation restores the initial filament morphology inside the droplet (Fig. 2d,e and Supplementary Fig. 8). Using an optimized sequence design^21^, fast kinetics have been achieved. The assembly and disassembly processes take place within 10 min. To quantify the reversibility of the dynamic processes, the porosity *Φ* inside the droplets, which is a direct measure of the degree of filament assembly (see Methods), was evaluated after two consecutive strand-displacement reactions. As shown in Fig. 2f, the degree of polymerization ((1 − *Φ*) × 100%) decreases from 55.1 ± 14.6% to 19.2 ± 2.8% and then returns approximately to its initial value of 54.4 ± 4.4%. This demonstrates the excellent reversibility of the disassembly and assembly processes powered by DNA hybridization.

### Reversible assembly by aptamer–target interactions

**Fig. 3:**
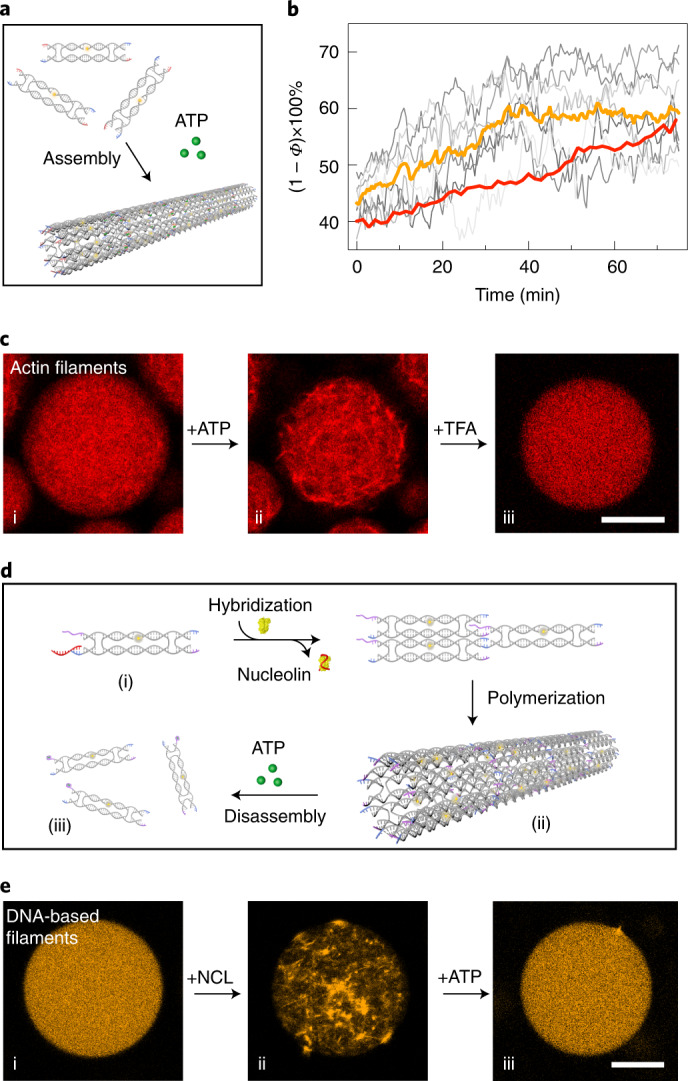
Comparison between DNA-based and actin filaments in cell-sized confinement. **a**, Schematic of polymerization of the DNA tiles containing split ATP aptamers upon addition of ATP. **b**, Normalized porosity ((1 − *Φ*) × 100%, corresponding to the degree of polymerization) in seven individual droplets (grey) and average polymerization for the DNA-based filaments (orange) and the actin filaments (red) over time during the polymerization processes. The degree of polymerization for the DNA-based filaments inside the droplets increases over time, until it reaches a dynamic steady state after 40 min. Actin filaments are polymerized at a comparable rate, reaching a similar degree of polymerization. **c**, Confocal microscopy images of droplets containing rhodamine-labelled actin filaments (*λ*_ex_ = 561 nm) (i) directly after encapsulation, (ii) 30 min after addition of ATP and (iii) after subsequent addition of TFA. The actin filaments are assembled on adding ATP and are disassembled after adding TFA. **d**, Schematic of the dual-responsive DNA tile containing an NCL-specific aptamer to trigger assembly and an ATP-specific aptamer to trigger disassembly of the filaments. **e**, Confocal microscopy images of droplets containing dual-stimuli-responsive Cy3-labelled filaments (*λ*_ex_ = 561 nm) (i) without NCL or ATP, (ii) after the addition of NCL and (iii) after the subsequent addition of ATP. The DNA-based filaments are assembled following the addition of NCL and subsequently disassembled after the addition of ATP. Scale bars, 20 μm. Source data

To integrate biologically relevant components into our synthetic system, we next regulated the assembly and disassembly of the DNA-based filaments by aptamer–target interactions. As shown in Fig. 3a, each DNA tile was functionalized with a split ATP aptamer^22,23^ with its two segments positioned on opposite ends of the tile. In the presence of ATP, the two aptamer segments can bind, leading to assembly of the filaments. The dynamic polymerization process inside the individual droplets was tracked and quantified by confocal microscopy, as shown in Fig. 3b (Supplementary Video 2 and Supplementary Fig. 9). After ~40 min, the growth of the DNA-based filaments inside the compartments reached a reaction plateau. The ATP aptamer has a lower affinity to ATP compared to the affinity between the toehold and the invader/anti-invader^24^. This means that higher concentrations of ATP are needed to achieve similarly fast reaction kinetics. Filament assembly within 10 min requires only 37.5 μM anti-invader (Supplementary Fig. 8). However, 2 mM ATP is needed to reach the steady state for ATP-triggered polymerization after ~40 min, as shown in Fig. 3b. To provide a direct comparison between the synthetic and natural cytoskeletons, we also tracked the ATP-triggered polymerization of rhodamine-labelled actin filaments inside individual droplets over time (Supplementary Video 3). The DNA-based filaments and the actin filaments bear close resemblance in terms of the dynamics of the time-resolved polymerization processes (Fig. 3b). More specifically, the (1 − *Φ*) × 100% values start at 43.3% (orange) and 40.1% (red) for the DNA-based and actin systems, respectively. Both systems reach their polymerized states within 75 min and the corresponding (1 − *Φ*) × 100% values change to 59.2% and 59%, respectively. The actin polymerization inside compartments can be reversed by adding trifluoroacetic acid (TFA, Fig. 3c) to the oil phase, inducing actin depolymerization^25^. In parallel, for the DNA-based filaments, each DNA tile is functionalized with two different aptamers, which can bind to nucleolin (NCL) and ATP targets, respectively. This enables dual-responsive DNA-based filaments, which can be assembled and disassembled in the presence of NCL and ATP, respectively, as depicted in Fig. 3d and verified by atomic force microscopy (Supplementary Fig. 10). The confocal microscopy images in Fig. 3e demonstrate the assembled and disassembled states within the cell-sized confinement following the addition of NCL and ATP directly before encapsulation, respectively. Supplementary Video 4 shows the dynamics of the polymerized filaments after the addition of NCL. A comparison between Fig. 3c and Fig. 3e reveals similar network morphologies of the DNA-based and actin filaments despite the fundamentally different building blocks involved. Another important feature of natural cytoskeletal elements is their directional growth from one end. We mimicked this behaviour by implementing seeded growth of the DNA-based filaments using a DNA origami segment as the nucleation seed (Supplementary Figs. 11−13 and Supplementary Table 1)^26–28^.

### Cargo transport along DNA filaments

**Fig. 4:**
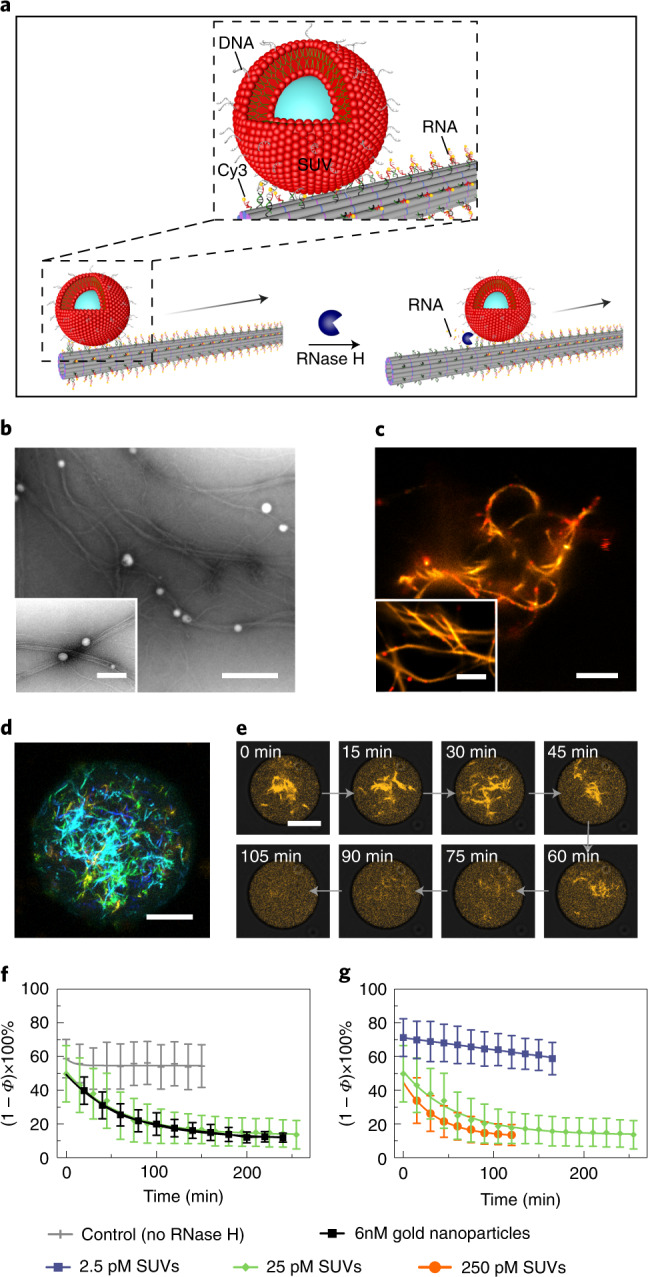
Proposed directional cargo transport guided along a DNA-based filament. **a**, Schematic of suggested cargo translocation by rolling along the DNA-based filament powered by RNase H-mediated hydrolysis. **b**, TEM images of the SUVs attached to the DNA-based filaments via cholesterol-tagged DNA. Scale bars, 500 nm and 200 nm (inset). **c**, STED images of the DNA–SUV networks. Scale bars, 5 μm and 2 μm (inset). **d**, Colour-coded *z*-projection of a DNA filament network in the presence of SUVs encapsulated into cell-sized confinement. Scale bar, 20 μm. **e**, Representative confocal time series of the DNA-based filaments within a water-in-oil droplet from *t* = 0 min to 105 min in time intervals of 15 min. The filaments lose their fluorescence over time due to the RNase H-mediated transport of SUVs (25 pM), while the fluorophores are successively cleaved along the DNA-based filaments. **f**, Porosity ((1 − *Φ*) × 100%) corresponding to the SUV transport in droplets over time without RNase H (grey), with gold nanoparticles (6 nM, black) and SUVs (25 pM, green). **g**, Porosity ((1 − *Φ*) × 100%) corresponding to the SUV transport in droplets over time at different SUV concentrations. The more SUVs are bound to the DNA filaments, the faster they lose their fluorescence, as more SUVs are transported along them. Error bars correspond to the standard deviation of *n* = 4–19 droplets. Scale bar, 10 μm. Source data

Finally, we set out to explore guided directional cargo transport along the DNA-based filaments, taking direct inspiration from the active vesicle transport by cytoskeletal motor proteins along microtubules within cells^29^. As depicted in Fig. 4a, the DNA-based filaments were modified with RNA overhangs to serve as transport tracks. The cargo was fully decorated with complementary DNA, such that the choice of cargo was versatile. For example, the cargo could be organic, such as small unilamellar vesicles (SUVs), or inorganic, such as gold nanoparticles. The SUVs were prepared from phosphatidylcholine (DOPC) lipids, with a mean hydrodynamic diameter of 65 ± 16 nm, as determined by dynamic light scattering (Supplementary Fig. 14), to mimic transport vesicles on microtubuli^29^. They were functionalized with cholesterol-tagged DNA and attached to the filaments by means of complementary base-pairing with multiple RNA overhangs on the filaments. We propose that the guided directional movement of the vesicle is based on a burnt-bridge mechanism^30–33^. On addition of RNase H, which selectively cleaves RNA in DNA–RNA hybrids, the hybridized RNA is hydrolysed. This promotes the rolling of the vesicle along the filament through hybridization of new single-stranded RNA further along the track with the DNA, which is abundantly coated on the vesicle. Because the DNA on the vesicle remains intact but the RNA track is depleted at the rear of the vesicle, this would impose transport of the vesicle guided by the DNA. The successful binding of SUVs to the DNA-based filaments was verified by the transmission electron microscopy (TEM) images in Fig. 4b. In addition, the stimulated emission depletion (STED) images in Fig. 4c corroborate attachment of Atto633-labelled SUVs to the filaments (Supplementary Fig. 15). Subsequently, the SUV–DNA networks were encapsulated in 3D confinement in the presence of RNase H. The colour-coded *z*-projection image in Fig. 4d was processed from a representative droplet and it nicely reveals SUV–DNA filament networks with great depth of field in 3D confinement. To optically disseminate the transport, each RNA overhang was modified with a fluorophore. The fluorophores are successively cleaved from the filament while the SUV rolls directionally along the track. Hence, the dissociation of the fluorophores from the filaments, which eventually leads to their homogeneous distribution inside the compartment, indicates active SUV transport, as demonstrated in Fig. 4e (Supplementary Video 5). The porosity inside the confinement of individual droplets was monitored over time using confocal microscopy (Supplementary Figs. 16 and 17) to evaluate the transport kinetics. It is noteworthy that we have previously used porosity to quantify filament assembly by attaching the fluorophore directly to one of the constituent strands in a DNA tile. In the current case, the fluorophore was positioned on the cleavable RNA–DNA chimaeric strand. Therefore, a decrease in (1 − *Φ*) × 100% is correlated with active SUV transport rather than filament disassembly. We released the contents from the droplets after cargo transport and confirmed, using TEM, that the DNA-based filaments remained intact (Supplementary Fig. 18). This proves that the decrease in (1 − *Φ*) × 100% is due to cleavage of the fluorophores from intact filaments, but not filament disassembly. The average transport velocity was estimated to be in the range of hundreds of nanometres per minute at an SUV concentration of 25 pM (Supplementary Note 1). A control experiment without RNase H proved that bleaching of the fluorophores only contributes 2% to the apparent decrease in (1 − *Φ*) × 100% (Fig. 4f and Supplementary Fig. 19a). We also performed an additional control experiment to evaluate the contributions of unspecific cutting of RNase H, showing negligible effects (Supplementary Fig. 19b).

Importantly, this proposed transport mechanism is not limited to biological cargo. A similar strategy was utilized to attach inorganic gold nanoparticles (20 nm in diameter) as cargo to the DNA-based filaments. The nanoparticles were functionalized with DNA, which binds to the sequence-complementary RNA overhangs (Supplementary Fig. 20), and experiments suggest that they roll along the droplet-encapsulated DNA filaments in the presence of RNase H (Supplementary Fig. 21). To provide further evidence for the guided rolling mechanism, the RNA overhangs were modified with biotin-streptavidin in this case so that the progressive cleavage along the filament could be visualized and confirmed by TEM (Supplementary Fig. 21). If the free DNA on the cargo is deactivated by hybridization with blocking DNA strands (Supplementary Fig. 22a), the rolling motion will be inhibited, whereas hopping or gliding motion could still take place. Importantly, the porosity measurements inside confinement show no decrease in the presence of the blocking strands after the addition of RNase H, neither for the gold nanoparticles nor for the SUVs (Supplementary Fig. 22b). The combined data thus suggest that transport takes place via cargo rolling along the DNA filaments. The cargo transport halts if the rolling motion is inhibited. It is noteworthy that the quenching effects from the gold particles in principle would facilitate the fluorescence decrease process. However, the transport velocity of the gold nanoparticles (6 nM) seems to be significantly lower than that of SUVs (25 pM) when comparing two systems that exhibit similar porosity change dynamics (Fig. 4f). In agreement with previous work^31^, we find that a denser DNA coating on the particle generally leads to faster motion (here 0.10 ± 0.01 strands per nm^2^ for gold nanoparticles versus 0.18 ± 0.01 strands per nm^2^ for SUVs, as determined by UV spectrophotometer measurements; Supplementary Figs. 23 and 24). The proposed rolling mechanism relies on the DNA strands on the cargo to continuously search for new RNA overhangs on the track for binding. Moreover, lipid vesicles differ fundamentally from inorganic particles, because their membranes are diffusive. This probably promotes hybridization of the cholesterol-anchored DNA strands on the lipid vesicles with the RNA overhangs on the filaments. These characteristics highlight the advantage of lipid vesicles as efficient cargo transport carriers in synthetic cells. Crucially, the overall cargo transport rate can be tuned by a set of experimental parameters, such as the concentration of RNase H (Supplementary Fig. 25) as well as the concentration ratio between cargo and filaments. More specifically, the decay constant increases from negligible decay for 2.5 pM SUVs to 0.017 ± 0.001 min^−1^ for 25 pM SUVs and 0.028 ± 0.001 min^−1^ for 250 pM SUVs (Supplementary Fig. 26). This indicates that a higher concentration of SUVs leads to a faster decrease in network fluorescence (Fig. 4g). Unlike previous reports, in which DNA origami or inorganic particles rolled on a surface^30–33^, our data suggest that the cargo transport in our case is guided along a linear filament track within 3D confinement. A key challenge is that SUVs tend to fuse with surfaces, especially in the presence of Mg^2+^, which is often required to stabilize DNA nanostructures. Here, droplet encapsulation provides an elegant solution. Because lipid vesicles are not only hollow containers but also the typical cargo carried by motor proteins for intracellular transport in living cells, their integration into DNA-based systems outlines an exciting route with rich opportunities in pursuit of creating synthetic cells from the bottom up.

## Discussion

Living cells possess a remarkable integral organization featuring transport and communication among distant components within a cell. In recent years, structurally similar replicas of some of these natural architectures have been constructed de novo from DNA. Although mere geometry is relatively straightforward to emulate thanks to the rapid advances in DNA nanotechnology, the realization of functional, and particularly multi-functional mimics remains an exciting challenge in the path towards the bottom-up construction of synthetic cells. DNA nanotubes are a nice example of the trajectory from structure^17^ to function^12–14^, recently progressing towards mimics of cytoskeletons^16,18^. Our study has outlined DNA-based cytoskeleton mimics and their operation in cell-sized confinement. Such filaments can undergo dynamic assembly and disassembly driven by biologically relevant molecules, such as ATP or engineerable synthetic triggers, including DNA fuel strands or aptamer–target interactions. After encapsulation, the sequential addition of molecules could be achieved by using microfluidic picoinjection^34^, fusion^35^ or light-triggered release of caged compounds^36,37^. The filaments further support apparent guided cargo transport along the filamentous tracks. As cargo we chose inorganic gold nanoparticles and lipid vesicles, inspired by the vast technological possibilities on the one hand and by the biological counterpart on the other. Our proposed DNA-based cargo transport takes place guided along a track, with the cargo travelling several tens of micrometres within tens of minutes. A challenging but insightful experiment would be monitoring the cargo transport along the DNA-based filaments on the single cargo level within the compartment. The transport rate of vesicles on microtubules in living cells is still much faster^38^. Here, it will be interesting to integrate other very recent mechanisms for transport along^39^ and in (ref. ^40^) DNA nanotubes. Our work thus stimulates ambition for future research, in which DNA-based systems could approach or even surpass the capabilities of nature. It will be of particular interest to engineer dynamic instability and filament polarity towards active force-generating DNA filaments. Along the route, we may engineer synthetic cells at the interface between technology and biology for applications in biomedicine, robotic drug delivery, nanomachinery, artificial cellular signalling and communication and beyond.

## Methods

### DNA tile design and assembly

The tile design and sequences in this study were adopted from ref. ^17^ with minor revisions. DNA tiles for all the presented systems were prepared as follows. Each DNA tile strand was mixed at a final concentration of 5 μM in a Tris-EDTA (TE)/Mg^2+^ buffer (10 mM Tris, 1 mM EDTA, 12 mM MgCl_2_, 5 mM NaCl, pH 8). A 100-μl solution was annealed using a thermocycler (Eppendorf) by heating the solution to 90 °C, and cooling it to 25 °C at a constant rate of 0.18 °C min^−1^ for a 6-h period. For the assembly of gold nanoparticles and filaments, 10 nM gold nanoparticles were mixed with 5 μM DNA filaments at room temperature and incubated overnight. All DNA strands were purchased from Sigma Aldrich and RNA–DNA conjugate strands from Integrated DNA Technologies. The DNA sequences for all DNA-based filament designs are provided in Supplementary Table 1.

### TEM

For TEM imaging of the DNA-based filaments, 10 μl of 100 nM DNA tiles were deposited on freshly glow-discharged carbon/formvar TEM grids. Before depositing the DNA tile solution, the grids were treated by negative glow discharge for 1 min. After 10 min of deposition, the grids were treated with a uranyl formate solution (2%) for 15 s.

### AFM

A 20-μl volume of 100 nM DNA-based filaments was deposited onto freshly cleaved mica (Ted Pella) and left to adsorb for 20 min, then 100 μl of buffer (1× TE/Mg^2+^) was added on top of the sample and the sample was imaged in fluid tapping mode using an atomic force microscope (Molecular Imaging, Bruker Technologies) with ScanAsyst In Fluid+ (Veeco Probes).

### Confocal fluorescence microscopy

A confocal laser scanning microscope LSM 880 or LSM 900 (Carl Zeiss) was used for confocal microscopy imaging. The pinhole aperture was set to one Airy unit and the experiments were performed at room temperature, if not stated otherwise. The images were acquired using a ×20 (Plan-Apochromat ×20/0.8 Air M27, Carl Zeiss) or ×63 objective (Plan-Apochromat ×63/1.4 oil DIC M27). Images were analysed and processed with ImageJ (NIH, brightness and contrast adjusted).

### Formation of surfactant-stabilized droplets

As previously described^41^, microfluidic polydimethylsiloxane-based (Sylgard 184, Dow Corning) devices for the formation of water-in-oil droplets were produced and assembled. The device layout of a single inlet device as used for encapsulation of the DNA filaments is shown in Supplementary Fig. 6. For the oil phase, 1.4 vol% of perfluoropolyether-polyethylene glycol (PEG) block-copolymer fluorosurfactants (PEG-based fluorosurfactant, Ran Biotechnologies) dissolved in HFE-7500 oil (DuPont) was used. The aqueous phase contained the encapsulated content and was varied as described in the corresponding sections. The fluid pressures were controlled by an Elveflow microfluidic flow control system. The fluids were injected into the channels via polytetrafluoroethylene tubing (0.4 × 0.9 mm, Bola). To observe the production process, an Axio Vert.A1 (Carl Zeiss) inverse microscope was used. As an alternative to the microfluidic formation of droplets, the aqueous phase was layered on top of the oil phase within a microtube (Eppendorf) and droplet formation was induced by manual shaking as described previously^42^.

### Polymerization of the ATP-sensitive DNA tiles

DNA tiles were stored in Tris-acetate-EDTA (TAE; 40 mM Tris, 20 mM acetic acid, 1 mM EDTA) at pH 8 containing 20 mM MgCl_2_. For instant polymerization into filaments, 500 nM DNA tiles were mixed with 10 mM ATP and encapsulated via microfluidics into surfactant-stabilized droplets. To visualize the polymerization process, 1 μM DNA tiles were mixed with 2 mM ATP and immediately encapsulated into droplets via the shaking approach. The reduced DNA to ATP ratio resulted in slower polymerization kinetics. The droplets were imaged directly after the encapsulation to monitor the process of filament formation over time inside individual droplets.

### Assembly and disassembly of the DNA tiles via aptamer–target interactions or strand-displacement reactions

For the aptamer-specific assembly, 500 nM DNA tiles were mixed with 1.5 μM nucleolin (Sigma Aldrich, cat. no. N2662) in 1× TAE buffer containing 20 mM MgCl_2_. For subsequent disassembly of the DNA filaments, 10 mM ATP was added to the solution. In the case of the strand-displacement-mediated (de-)polymerization, 500 nM DNA tiles were mixed with 10 μM invader strands and encapsulated into droplets immediately afterwards, which induced the disassembly of the DNA filaments. By addition of 37.5 μM anti-invader strands directly before encapsulation, the filaments were reassembled.

### Actin encapsulation

Actin (purified from acetone powder from New Zealand white rabbit skeletal muscle, based on the method of ref. ^43^, modified after ref. ^44^) was stored in so-called GAB buffer containing 2 mM Tris/HCl, pH 8, 0.2 mM CaCl_2_, 0.2 mM ATP, 0.005% NaN_3_ and 0.2 mM dithiothreitol (DTT), at −80 °C. The actin monomers were labelled with methanol-dissolved rhodamine-phalloidin (Biotium) by mixing 20 μl actin with 20 μl AB DTT DD (double-density) buffer (50 mm HEPES, pH 7.4, 50 mM KCl, 8 mM MgCl_2_, 20 mM EGTA, 20 mM DTT) and with 3.3 μl 10× actin polymerisation buffer (20 mM Tris-HCl, pH 8, 500 mM KCl, 20 mM MgCl_2_, 10 mM NaATP). Subsequently, 13 μl of rhodamine-phalloidin (13 U) were added to the solution, which was immediately encapsulated into droplets and imaged during polymerization.

### Analysis of the degree of polymerization

To analyse the degree of polymerization for DNA-based and actin filaments, images were thresholded using Otsu’s method. For each droplet, a circular area of 133 μm^2^ in the droplet centre was chosen and the relative amount of fluorescent pixels was analysed using the image analysis tool in ImageJ. The degree of polymerization was defined via the porosity *Φ* as (1 − *Φ*) × 100% = (1 − *A*_empty_/*A*_total_) × 100% = *A*_filament_/*A*_total_ × 100%. Here, *A*_empty_ is the void area, *A*_total_ is the total area and *A*_filament_ is the area that is occupied by the DNA filaments. This corresponds to the degree of polymerization of the DNA tiles inside the droplets.

### Preparation of the DNA origami seeds

The seed consisted of a layer of 12 helices modified with staples that connect the first and twelfth helix to form a hollow cylinder. The designed nanotube seed consisted of a single-stranded M13mp18 scaffold (Tilibit Nanosystems), 72 short staple strands, six capture strands and 24 adapter strands (for DNA sequences, see Supplementary Table 1; for the strand routing diagram, see Supplementary Fig. 11). The mixture was annealed in a ratio of 1:10:10:10 for scaffold, capture strands, adapter strands and staple strands, respectively. All samples were assembled in TAE buffer (40 mM Tris-acetate, 1 mM EDTA) with 12.5 mM MgCl_2_ by slowly cooling it from 90 °C to 25 °C over a 3-h period. The product was then purified by spin filtration with a 100-kDa molecular weight cutoff (MWCO) filter (Amicon, Millipore) to remove the extra staple strands, adapter strands and capture strands.

### Functionalization of the gold nanoparticles with DNA

Tris(2-carboxyethyl)phosphine (200 mM, 1 h) was used to reduce thiol-modified oligonucleotides (5′-GAC ACT AAC TAA TGA TTT-Thiol-3′ from IDT, HPLC purified) in water. Thiol-modified oligonucleotides and gold nanoparticles (20-nm diameter, Sigma Aldrich) were then incubated at a molar ratio of DNA to particles of 2,000:1 in a 0.5× Tris Borate EDTA (TBE) buffer solution for 20 h at room temperature. The concentration of NaCl was slowly increased to 500 mM to increase the thiolated DNA density on the particles. The particle–DNA conjugates were then washed using a 0.5× TBE buffer solution in 100-kDa (MWCO) centrifuge filters to remove the free oligonucleotides. The concentration of gold nanoparticles was measured at 520 nm (extinction coefficient = 9.21 × 10^8^ M^−1^ cm^−1^) using a spectrophotometer (Eppendorf).

### Quantification of DNA density on gold nanoparticles

The density of the DNA strands on the gold nanoparticles (Supplementary Fig. 23) was quantified by releasing the DNA from the gold nanoparticles and measuring the released DNA concentration using UV–vis spectroscopy (Eppendorf) according to a protocol adapted from ref. ^45^. Specifically, 0.5 ml of 6 nM DNA-modified gold nanoparticles with 1× TBE buffer was prepared. Its concentration was measured by UV–vis spectroscopy at the absorbance maximum of 520 nm, with an extinction coefficient of *ϵ*_AuNP_ = 9.21 × 10^8^ M^−1^ cm^−1^. The DNA on the particles was released by adding 20 μl of a 1 M DTT solution. The mixture was incubated with DTT for 2 h to ensure complete dissolution and then centrifuged at a speed of 8,500 r.c.f. for 30 min. The supernatant was carefully collected and then measured using UV–vis spectroscopy (at the absorbance maximum of 260 nm, extinction coefficient *ϵ*_DNA_ = 180,400 M^−1^ cm^−1^) to calculate the concentration of DNA released from the particles. The amount of DNA on the gold nanoparticles was calculated using (*A*_260_/*ϵ*_DNA_)/(*A*_520_/*ϵ*_AuNP_). *A*_260_ and *A*_520_ are the absorbance values at 260 nm and 520 nm, respectively. The obtained result was divided by the surface area of the 20-nm gold nanoparticle to yield the DNA density per gold nanoparticle, which gave rise to 0.10 ± 0.01 DNA strands per nm^2^.

### Attachment of gold nanoparticles on the DNA origami seeds

Purified DNA origami seeds were mixed with DNA-functionalized gold nanoparticles in a ratio of 1:5 and then annealed from 35 °C to 25 °C for 12 h. The annealed product of the seeds with gold nanoparticles was purified by agarose gel electrophoresis (running buffer, 0.5 × TBE with 11 mM MgCl_2_; voltage, 15 V cm^−1^; running time, 1 h). Selected bands were cut out and the seeds with gold nanoparticles were extracted from the gel in Freeze-Squeeze columns (Bio-Rad) at 4 °C. The gold-nanoparticle-labelled seeds were then imaged with TEM (Supplementary Fig. 12).

### Seeded growth of the DNA-based filaments

Purified DNA origami seeds and gold nanoparticles were mixed with two different tiles (Supplementary Table 1) in a ratio of 1:1,000:1,000 and were then incubated at 32 °C for 12 h. After incubation, the seeded filaments were imaged with TEM or encapsulated into water-in-oil droplets (Supplementary Fig. 13).

### STED imaging

DNA filaments and SUVs were imaged on an Abberior expert line (Abberior Instruments) with a pulsed STED line at 775 nm using excitation lasers at 560 nm and 640 nm and spectral detection. Detection windows were set to 650–725 nm and 580–630 nm to detect Atto633-labelled SUVs and Cy3-labelled DNA filaments, respectively. Images were acquired with a ×100/1.4 NA oil immersion lens (Olympus). The pixel size was set to 30 nm and the pinhole was set to 1 AU. Atto633 and Cy3 were imaged semi-simultaneously during a first acquisition with STED at 775 nm. Images were analysed and processed with ImageJ (NIH; brightness and contrast adjusted).

### SUV extrusion

SUVs composed of 99% 18:1 DOPC (1,2-dioleoyl-*sn*-glycero-3-phosphocholine) and 1% Atto633-DOPE (Atto633–1,2-dioleoyl-*sn*-glycero-3-phosphoethanolamin) were formed by mixing the lipids dissolved in CHCl_3_ in a glass vial and subsequent solvent evaporation under a stream of nitrogen gas. The glass vial was then placed under a vacuum for 30 min to remove residual traces of solvent. Afterwards, the lipids were resuspended in phosphate buffered saline pH 7.4 (Thermo Fisher) at a final concentration of 1 mM lipids. The solution was vortexed for 10 min to trigger liposome formation. SUVs were then formed by extruding the liposome solution 13 times through a polycarbonate filter with a pore size of 50 nm (Avanti Polar Lipids). The SUVs were stored at 4 °C until use.

### Quantification of the DNA density on SUVs

To quantify the DNA density on SUVs (Supplementary Fig. 24), we first measured the amount of lipids after SUV extrusion. The fluorescence intensity of the lipid mixture (99% DOPC, 1% Atto633-DOPE) was determined before and after extrusion with a microplate reader (Spark, Tecan). This revealed that 9.07% of the lipids are lost during the extrusion process (mean and standard deviation from *n* = 4 independent measurements). Subsequently, we determined the incorporation efficiency of single-stranded cholesterol-tagged DNA into SUVs. For this, the concentration of DNA was quantified from UV–vis absorbance measurements with a spectrophotometer (Nanophotometer, Implen). A reference measurement was taken before addition of the DNA to SUVs. Afterwards, the cholesterol-tagged DNA was incubated in excess with SUVs for 10 min, then the SUVs were centrifuged at 100,000*g* for 1 h (Optima Ultracentrifuge, Beckman Coulter). The supernatant was extracted carefully and the DNA concentration in the supernatant was measured with UV–vis spectroscopy. This corresponds to the unbound fraction of DNA and showed that 27.8 ± 2.2% of 2 μM cholesterol-tagged DNA binds to SUVs (10 μM lipids before extrusion). Taken together, this leads to a DNA density of 0.18 ± 0.01 DNA strands per nm^2^.

### SUV transport

For the transport experiments, SUVs were incubated for 2 min with cholesterol-tagged DNA (5′-GAC ACT AAC TAA TGA TTT-Chol-3′) in a lipid-to-DNA ratio of 2.5:1. In the meantime, DNA filaments (final concentration, 250 nM) were mixed with 1× RNase H reaction buffer (50 mM Tris-HCl, 75 mM KCl, 3 mM MgCl_2_, 10 mM DTT, pH 8.3) and incubated for 2 min. Subsequently, DNA filaments and SUVs were mixed with 5 mM MgCl_2_. Finally, 0.25 U μl^−1^ of RNase H (final concentration, 50 mM KCl, 10 mM Tris-HCl, 0.1 mM EDTA, 1 mM DTT, 200 μg ml^−1^ BSA, 50% glycerol, pH 7.4; NEB) was added. Water-in-oil droplets were formed using the shaking method, put into an observation chamber and immediately observed using confocal microscopy for 2–6 h.

### Analysis of the SUV transport along the DNA filaments

To analyse vesicle transport along the DNA filaments, images were thresholded using Otsu’s method. For each droplet, a circular area in the droplet centre of 900 μm^2^ was chosen and the relative amount of fluorescing pixels was analysed. By analysing the porosity, a direct measure of the amount of SUV transport within the droplets was obtained.

### Gold nanoparticle transport

DNA tile strands and biotin RNA substrate were mixed and assembled at a final concentration of 1 μM. Then 1 μM streptavidin was mixed with the filaments and incubated at 4 °C for 4 h. After that, 10 nM gold particles with diameters of 20 nm were added to the mixed solution and incubated overnight at room temperature. For the transport, filaments were mixed with 1× RNase H reaction buffer first, then 0.1 U of RNase H was added. After 2 h, 10 μl of reaction solution was taken for TEM imaging.

## Online content

Any methods, additional references, Nature Research reporting summaries, source data, extended data, supplementary information, acknowledgements, peer review information; details of author contributions and competing interests; and statements of data and code availability are available at 10.1038/s41557-022-00945-w.

## Supplementary information

Supplementary Information Supplementary Note 1, Figs. 1–26, video captions 1–5.

Supplementary Table 1 Lists of DNA sequences.

Supplementary Video 1 Dynamics of the toehold-modified DNA filaments inside water-in-oil droplets.

Supplementary Video 2 Time-resolved polymerization of the ATP-aptamer-modified DNA filaments inside water-in-oil droplets.

Supplementary Video 3 Time-resolved polymerization of actin filaments inside water-in-oil droplets.

Supplementary Video 4 Dynamics of the DNA filaments with aptamers for nucleolin and ATP targets inside water-in-oil droplets.

Supplementary Video 5 SUV transport on DNA filaments inside water-in-oil droplets.

## Data Availability

Data supporting this study are available in the manuscript, Supplementary Information and are also available from the corresponding author on request. Source data are provided with this paper.

## Source data

Source Data Fig. 2 Statistical source data.
Source Data Fig. 3 Statistical source data.
Source Data Fig. 4 Statistical source data.
