# Supplementary material for: Functional DNA-based cytoskeletons for synthetic cells
Source: Nat Chem. 2022 Jun 20;14(8):958–63. doi: 10.1038/s41557-022-00945-w (PMC9359917; doi:10.1038/s41557-022-00945-w)
Supplement: Supplementary file 1 — Supplementary Note 1, Figs. 1–26, video captions 1–5. [file 41557_2022_945_MOESM1_ESM.pdf]

nature portfolio

https://doi.org/10.1038/s41557-022-00945-w

Supplementary information

Functional DNA-based cytoskeletons for synthetic cells

In the format provided by the
authors and unedited

Supplementary Information: Functional DNA-based cytoskeletons for synthetic cells

Pengfei Zhan[1,2§], Kevin Jahnke[3,4§*], Na Liu[1,2*], Kerstin Göpfrich[3,4*]

[1] 2nd Physics Insitute, University of Stuttgart,

Im Pfaffenwaldring 57, D-70569 Stuttgart, Germany

[2] Max Planck Institute for Solid State Research,

Heisenbergstraße 1, D-70569 Stuttgart, Germany

[3]Biophysical Engineering Group, Max Planck Institute for Medical Research,

Jahnstraße 29, D-69120 Heidelberg, Germany,

[4]Department of Physics and Astronomy, Heidelberg University,

D-69120 Heidelberg, Germany

[§]These authors contributed equally.

*

E-mail: kevin.jahnke@mr.mpg.de,na.liu@pi2.uni-stuttgart.de,kerstin.goepfrich@mr.mpg.de

Contents

Supplementary Notes **4**

Supplementary Note S1: Estimation of the SUV velocity along the DNA filaments 4

Supplementary Figures **6**

Supplementary Figure S1: Strand routing diagrams for the DNA tiles 6

Supplementary Figure S2: Assembly of the DNA filaments at different tile concentrations 7

Supplementary Figure S3: Assembly of the DNA filaments at different Mg^{2+} concentrations 8

Supplementary Figure S4: Assembly of the DNA filaments at different temperatures 9

Supplementary Figure S5: Confocal images of the DNA-based filaments in bulk . 10

Supplementary Figure S6: Microfluidic device and droplet formation 11

Supplementary Figure S7: AFM images of assembly and disassembly of the DNA-based filaments by strand displacement reactions 12

Supplementary Figure S8: Determination of the required concentrations of invader and anti-invader strands 13

Supplementary Figure S9: Low ATP concentrations do not induce filament assembly 14

Supplementary Figure S10: AFM images of the aptamer-specific assembly and disassembly of the DNA-based filaments 15

Supplementary Figure S11: Strand routing diagram for the DNA origami seed . . 16

Supplementary Figure S12: Seeded growth mechanism 17

Supplementary Figure S13: TEM and confocal images of seeded growth 18

Supplementary Figure S14: Size distribution of the SUVs 19

Supplementary Figure S15: STED imaging confirms the binding of the SUVs to the DNA-based filaments 20

Supplementary Figure S16: Color-coded z-projections of the DNA-SUV networks . 21

Supplementary Figure S17: Composite images of the DNA-SUV networks 22
Supplementary Figure S18: DNA filaments remain intact after transport 23
Supplementary Figure S19: Control experiments for SUV transport 24
Supplementary Figure S20: TEM images of the gold nanoparticles attached to the DNA filaments 25
Supplementary Figure S21: TEM indicates rolling of gold nanoparticles along the DNA-based filaments 26
Supplementary Figure S22: Mechanism of motion 27
Supplementary Figure S23: DNA density on gold nanoparticles 28
Supplementary Figure S24: DNA density on SUVs 29
Supplementary Figure S25: Influence of the RNase H concentration on the SUV transport 30
Supplementary Figure S26: Decay constants for the DNA network fluorescence decrease 31

Supplementary Videos **31**
Supplementary Video S1: Dynamics of the toehold-modified DNA filaments inside water-in-oil droplets 31
Supplementary Video S2: Time-resolved polymerization of the ATP-aptamer-modified DNA filaments inside water-in-oil droplets 32
Supplementary Video S3: Time-resolved polymerization of actin filaments inside water-in-oil droplets 32
Supplementary Video S4: Dynamics of the DNA filaments with aptamers for nucleolin and ATP targets inside water-in-oil droplets 32
Supplementary Video S5: SUV transport on DNA filaments inside water-in-oil droplets 33

References **34**

Supplementary Notes

Supplementary Note S1: Estimation of the SUV velocity along the DNA filaments

From the quantitative measurements of the porosity over time (see Fig. 4f, main text), we can provide a rough estimate for the transport velocity of the individual SUVs. For this purpose we need to first estimate 1) the total length of the DNA filaments inside a droplet and 2) the total number of the SUVs inside a droplet.

1) Each DNA tile has an approximate length of $l_t = 14\,\mathrm{nm}$, and the tubular filaments have a circumference of approximately 6 tiles.[1] Assuming that all tiles are polymerized into filaments, we can calculate the maximum total length of the DNA filaments per liter to be

$$l_{tot} = \frac{1}{6} \cdot c_t \cdot N_A \cdot l_t \approx 0.35 \cdot 10^9\,\mathrm{m/l}\,, \tag{1}$$

with c_t being the DNA tile concentration (250 nM) and N_A the Avogadro number.
With an average droplet diameter of 40 μm in this experiment and hence a droplet volume of approximately 40 pl, the total filament length per droplet amounts to $\approx 14\,\mathrm{mm}$.

2) We can calculate the SUV concentration from the total lipid concentration. We first compute the amount of lipids per SUV and from this the total number of SUVs per volume. The SUV diameter has been measured to be 65 nm. This yields

$$n_{\mathrm{Lipid}} = 2 \cdot \frac{4\pi r^2}{0.7\mathrm{nm}^2} \approx 38000\,\mathrm{lipids}\,, \tag{2}$$

where the area of each lipid is assumed to be $0.7\,\mathrm{nm}^2$ and the lipid bilayer is composed of two layers of lipids. Given a total lipid concentration of 10 µM as used in the experiment,

this leads to

$$n_{\mathrm{SUV}} = \frac{c_{\mathrm{Lipid}} \cdot N_A}{n_{\mathrm{Lipid}}} \approx 1.6 \cdot 10^{14}\,/\mathrm{l}\,. \tag{3}$$

This equals an SUV concentration of approximately 250 pM. With a droplet volume of 40 pl, we estimate that a total of 6400 SUVs have been encapsulated per droplet.

From Figure 4, we find that the filament fluorescence decreases with a half life of $t_{1/2} =$ 25 min for 250 pM SUVs. This means that 6400 SUVs roll on average over half of the total filament length in a droplet, hence 7 mm, during this time. A single SUV thus covers a distance of approximately 1 μm in 25 minutes, or $v_{\mathrm{SUV}} \approx$44 nm/min. Note that photobleaching only accounts for a 2 % decrease of the porosity (see Fig. S19a). This would result in a negligible increase of the calculated transport velocities, but its minor contribution has been disregarded here. This can be transformed into rotations of single SUVs via

$$v_{\mathrm{rot}} = \frac{v_{\mathrm{SUV}}}{2\pi r} \approx 0.22\mathrm{rot/min}\,. \tag{4}$$

All in all, this yields transport velocities for the different conditions:

Table 1: Estimation of the vesicle velocity along the DNA filaments.

lipid concentration	# SUVs per droplet	SUV concentration	half-life	velocity
0.1 µM	64	2.5 pM	636 min	170 nm/min
1 µM	640	25 pM	40 min	280 nm/min
10 µM	6400	250 pM	25 min	44 nm/min

Table 2: Estimation of the gold nanoparticle velocity along the DNA filaments.

nanoparticle concentration	# gold nanoparticles per droplet	half-life	velocity
6.4 nM	154200	44 min	1.03 nm/min

Supplementary Figures

Supplementary Figure S1: Strand routing diagrams for the DNA tiles

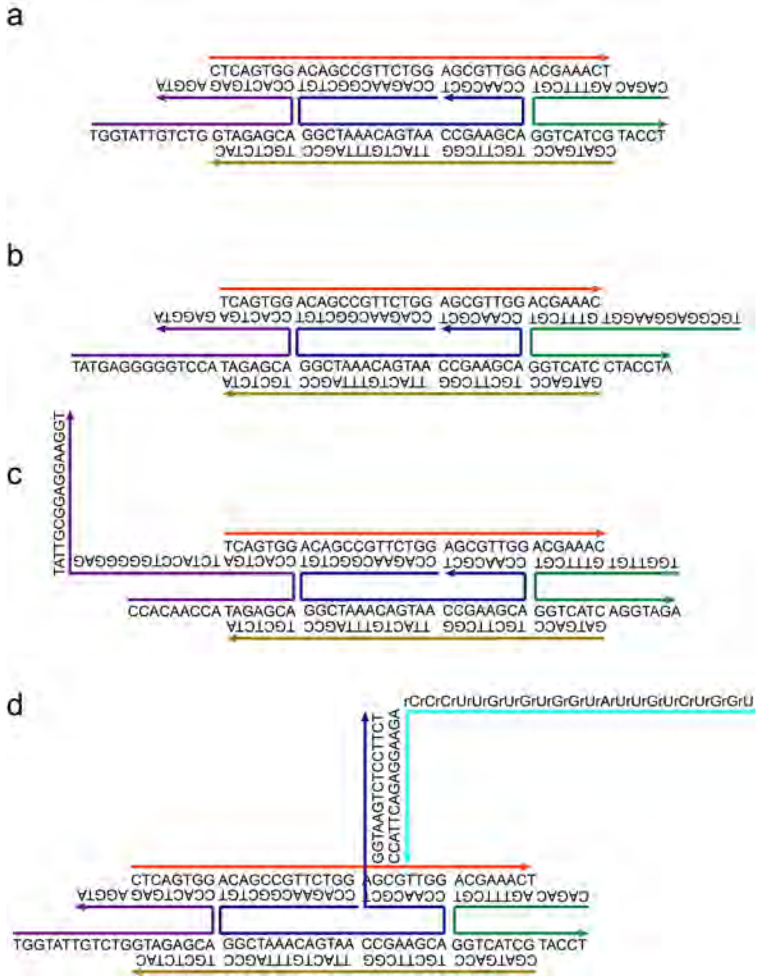

Figure 1: Strand routing diagrams for the DNA tiles. **a** DNA tile for the toehold strand displacement reaction in Fig. 2 according to Green et al.[2] **b** DNA tile for the ATP split-aptameric reaction in Figs. 3a and b. The ATP aptamer sequence was taken from Huizenga et al.[3] **c** DNA tile for the dual-aptamer reaction in Figs. 3d and e. **d** DNA tile for the SUV and gold nanoparticle transport reaction in Fig. 4. Overhang sequences for the rolling were adapted from Yehl et al.[4]

Supplementary Figure S2: Assembly of the DNA filaments at different tile concentrations

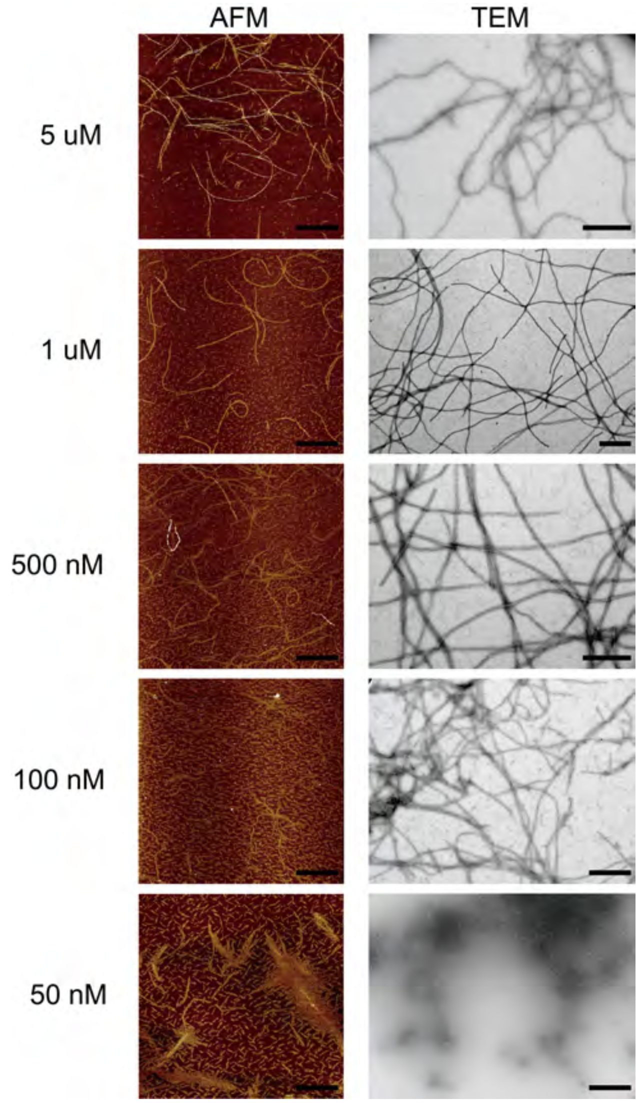

Figure 2: Assembly of the DNA filaments at different tile concentrations. AFM (left column) and TEM (right column) images of the formed DNA filaments annealed at DNA tile concentrations from 5 μM (top) to 50 nM (bottom). At a concentration of 5 μM used in the most experiments, a high yield of the correctly formed DNA filaments is obtained. Scale bars: 200 nm.

Supplementary Figure S3: Assembly of the DNA filaments at different Mg^{2+} concentrations

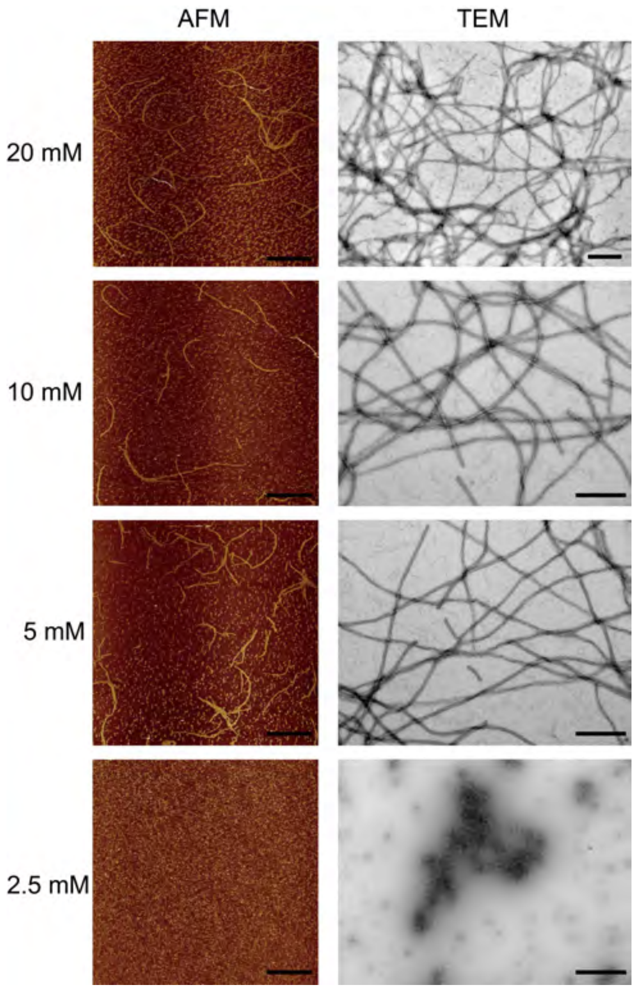

Figure 3: Assembly of the DNA filaments at different Mg^{2+} concentrations from 2.5 mM to 20 mM. AFM (left column) and TEM (right column) demonstrate successful DNA filament assembly at Mg^{2+} concentrations above 5 mM. Scale bars: 200 nm.

Supplementary Figure S4: Assembly of the DNA filaments at different temperatures

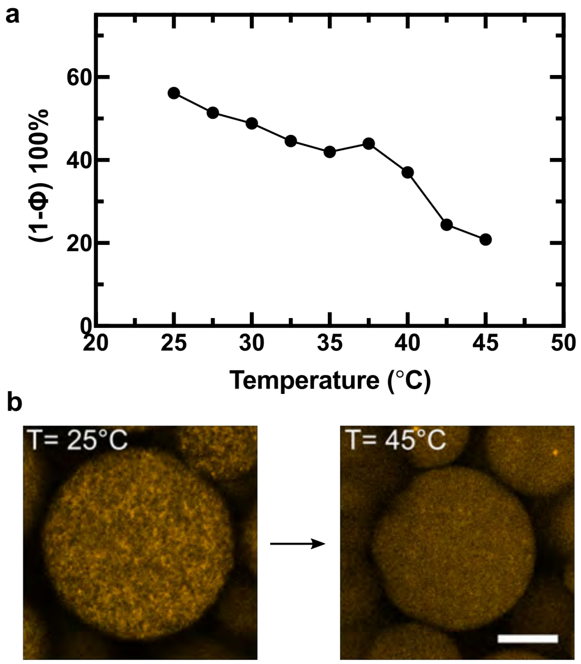

Figure 4: **a** Dependence of the porosity $(1-\Phi)100\%$ on temperature. **b** Confocal fluorescence microscopy images of DNA filaments at 25 °C and 45 °C, respectively. The filaments are completely disassembled at 45 °C, resulting in a homogeneous distribution of the fluorophores inside the droplet lumen. Scale bar: 20 μm.

Supplementary Figure S5: Confocal images of the DNA-based filaments in bulk

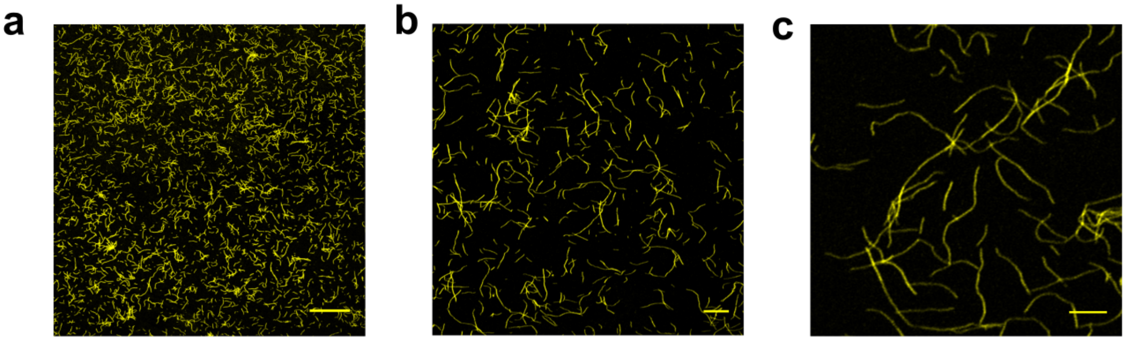

Figure 5: Confocal images of the DNA filaments in bulk. Cy3-labelled DNA filaments (λ_{ex} = 561 nm) in 1× TAE buffer containing 20 mM $MgCl_2$ at different magnifications. Due to Mg^{2+} mediated interactions, the DNA filaments are prone to attach to the glass coverslide. Scale bars: (**a**) 50 µm, (**b**) 10 µm and (**c**) 5 µm, respectively.

Supplementary Figure S6: Microfluidic device and droplet formation

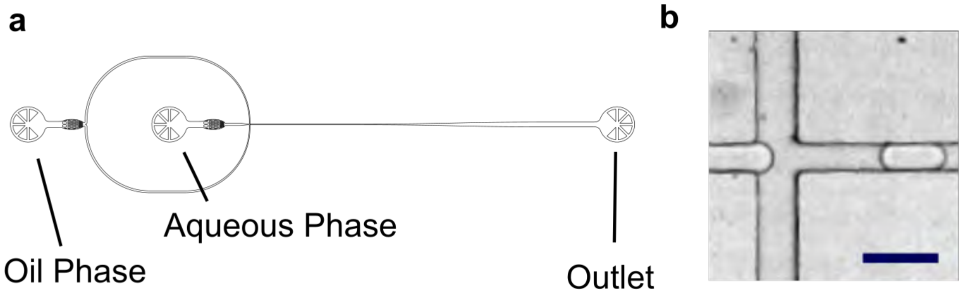

Figure 6: Microfluidic device and droplet formation. **a** Layout of the microfluidic T-junction device for the encapsulation of the DNA filaments (supplied via the aqueous phase) into surfactant-stabilized water-in-oil droplets. The droplets are collected from the outlet for further imaging. The microfluidic PDMS devices (Sylgard184, Dow Corning, USA) have been fabricated according to a previously published protocol [5] (see Methods). **b** Bright-field high-speed camera image of a flow-focusing T-junction during the droplet formation. Scale bar: 50 µm.

Supplementary Figure S7: AFM images of assembly and disassembly of the DNA-based filaments by strand displacement reactions

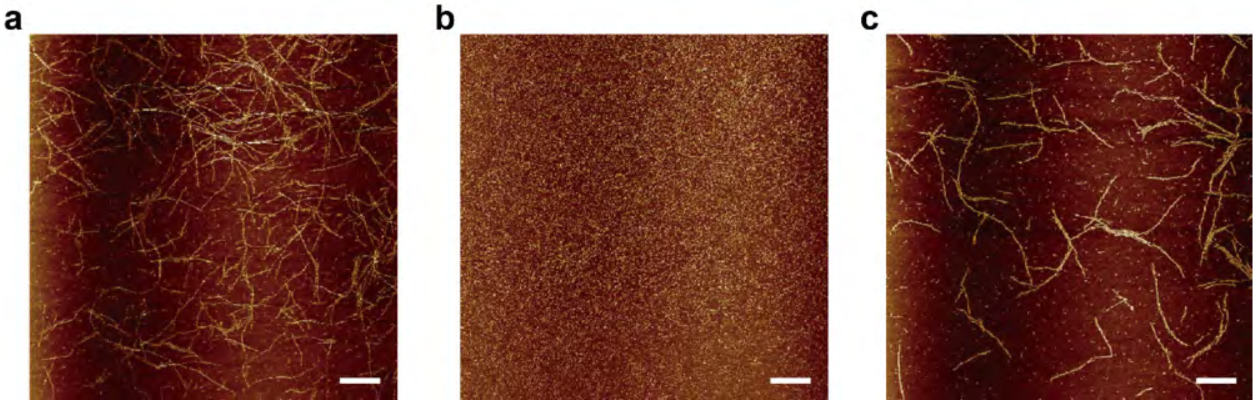

Figure 7: AFM images of reversible assembly and disassembly of the DNA filaments by subsequent strand displacement reactions.[2] **a** AFM image of the assembled DNA filaments before adding the invader strands. **b** AFM image of the disassembled DNA filaments after adding the invader strands. The DNA filaments are disassembled into individual DNA tiles. **c** AFM image of the re-assembled DNA filaments upon adding the anti-invader strands. Scale bars: 100 nm.

Supplementary Figure S8: Determination of the required concentrations of invader and anti-invader strands

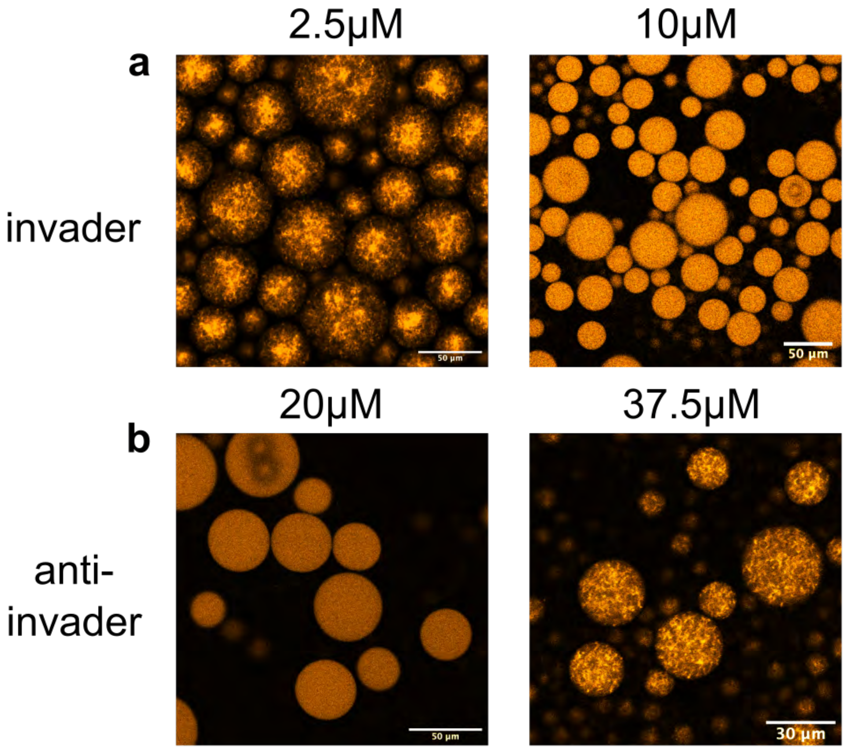

Figure 8: Determination of the required concentrations of invader and anti-invaders. **a** Confocal images of 1 µM Cy3-labelled DNA filaments (λ_{ex} = 561 nm) in 1× TAE buffer containing 20 mM $MgCl_2$. Addition of 2.5 μM invader strands is not sufficient to trigger the filament disassembly, whereas 10 μM can trigger the disassembly. **b** Confocal images of 1 µM Cy3-labelled DNA filaments (λ_{ex} = 561 nm) in 1× TAE buffer containing 20 mM $MgCl_2$. Addition of 20 μM anti-invader strand is not sufficient to trigger the filament assembly, whereas 37.5 μM can trigger the assembly. Images were taken approximately 10 minutes after droplet production. From these results, the higher concentrations have been chosen for the respective experiments.

Supplementary Figure S9: Low ATP concentrations do not induce filament assembly

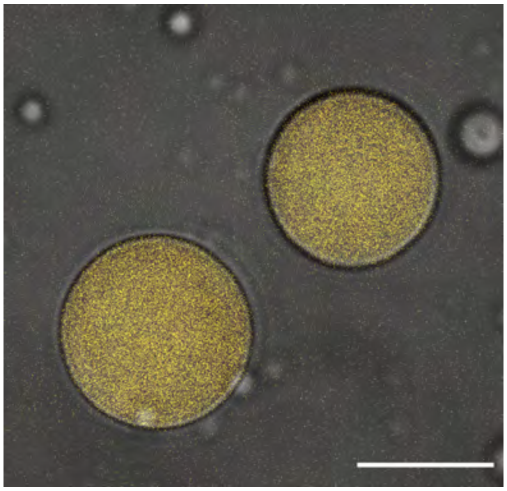

Figure 9: Low ATP concentrations do not induce filament assembly. Confocal microscopy image of 1 µM Cy3-labelled DNA tiles (λ_{ex} = 561 nm) in 1× TAE buffer containing 20 mM $MgCl_2$ and 1 mM ATP. DNA tiles are still distributed homogeneously within the water-in-oil droplets after about 10 minutes. Scale bar: 20 µm.

Supplementary Figure S10: AFM images of the aptamer-specific assembly and disassembly of the DNA-based filaments

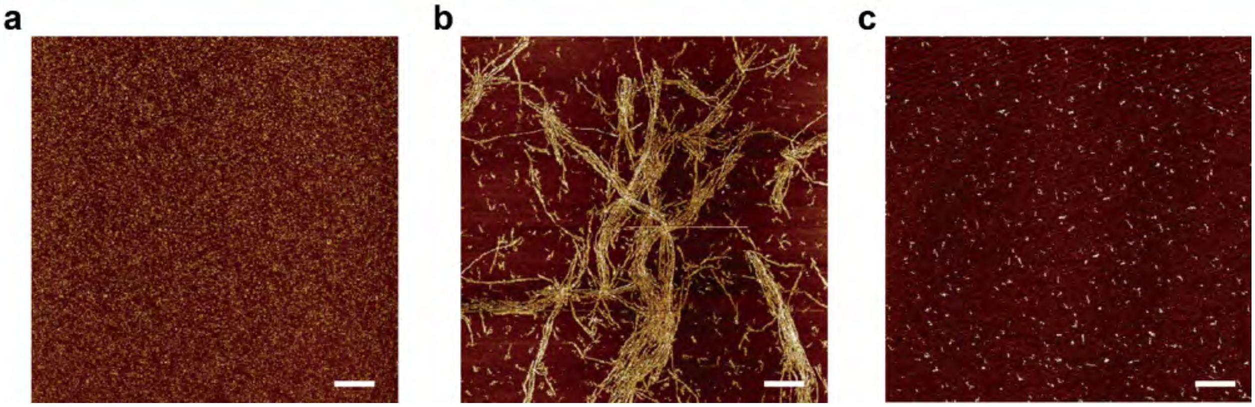

Figure 10: AFM images of the aptamer-specific reversible assembly and disassembly of the DNA filaments. **a** AFM image of the DNA tiles before adding NCL. Scale bar: 50 nm. **b** AFM image of the DNA filaments after adding NCL. DNA tiles are assembled into DNA filaments. Scale bar: 100 nm. **c** AFM image of the DNA tiles upon subsequent addition of ATP. DNA tiles are again disassembled. Scale bar: 50 nm.

Supplementary Figure S11: Strand routing diagram for the DNA origami seed

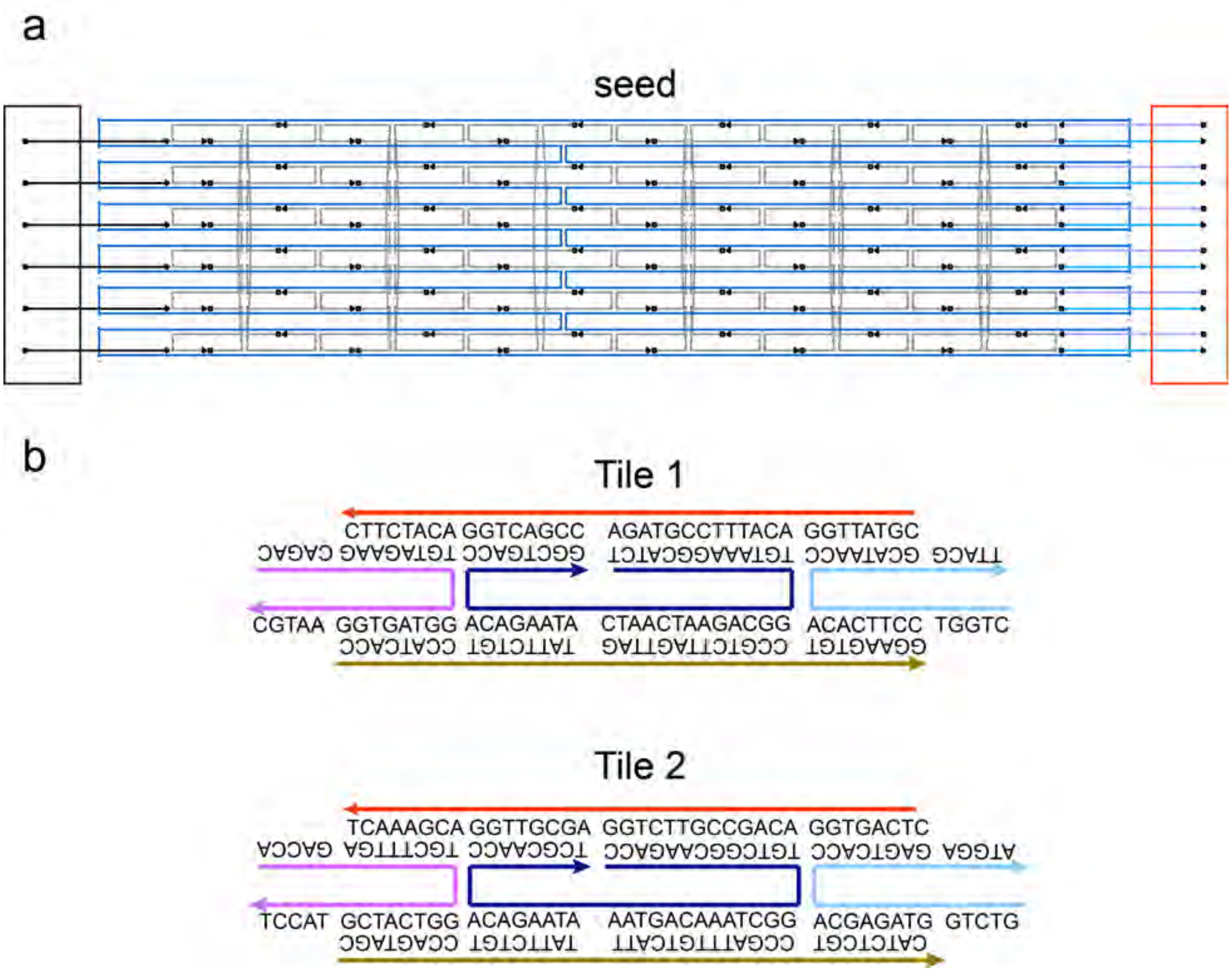

Figure 11: Strand routing diagram for the DNA origami seed to implement a seeded growth mechanism for the DNA filaments.[6] **a** Layout of the DNA origami seed with the DNA overhangs for the attachment of a gold nanoparticle (black box) and the DNA tiles (red box). **b** Design and sequences of the tiles, which are attached to the overhangs on the DNA origami seed for the directional growth of the filament. All DNA sequences are provided in Supplementary Dataset 1, adapted from Mohammed et al.[6]

Supplementary Figure S12: Seeded growth mechanism

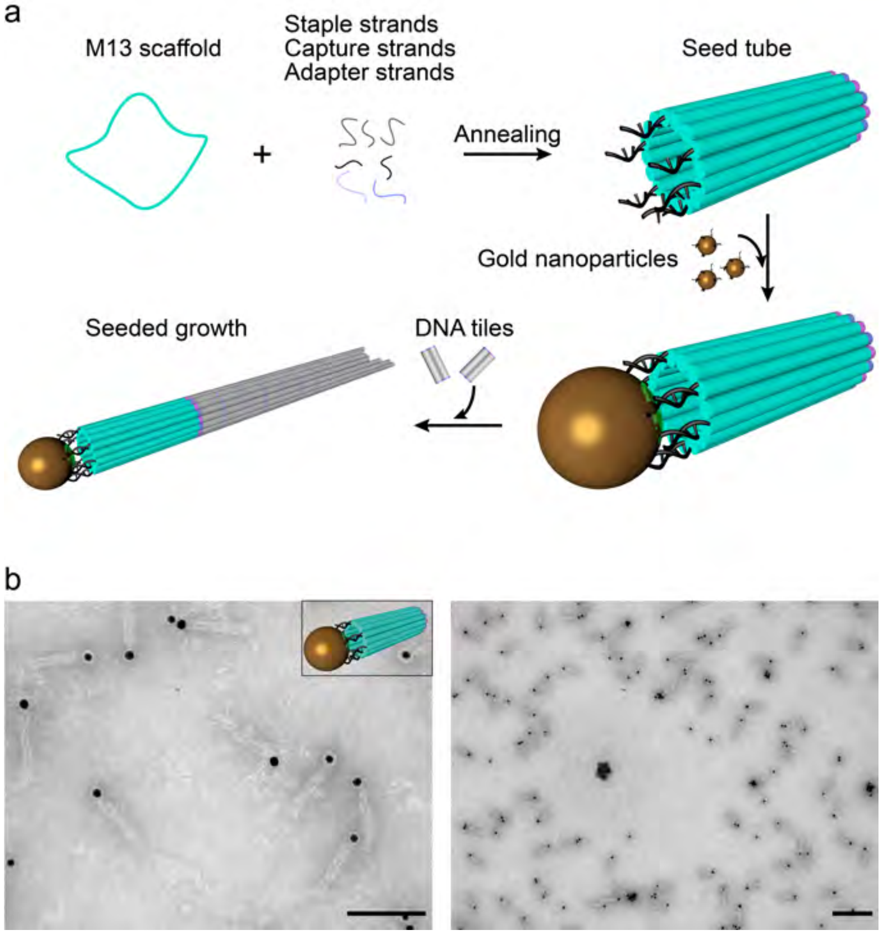

Figure 12: **a** Mechanism for the seeded growth of the DNA filaments.[6] The M13 scaffold is hybridized with specifically designed and custom-synthesized staple strands to form a hollow seed tube. A gold nanoparticle (10 nm diameter) is attached to one end of the tube via complementary base pairing. It serves as a label to distinguish the two ends of the seed for TEM imaging. The DNA tiles are added to the seed to implement seeded and directional growth of the DNA filaments. **b** TEM images of the DNA origami seeds. The gold nanoparticles are clearly visible on one of the ends of the tubes, leaving the other end open for attachment of the DNA tiles. Scale bars: 100 nm (left image) and 200 nm (right image), respectively.

Supplementary Figure S13: TEM and confocal images of seeded growth

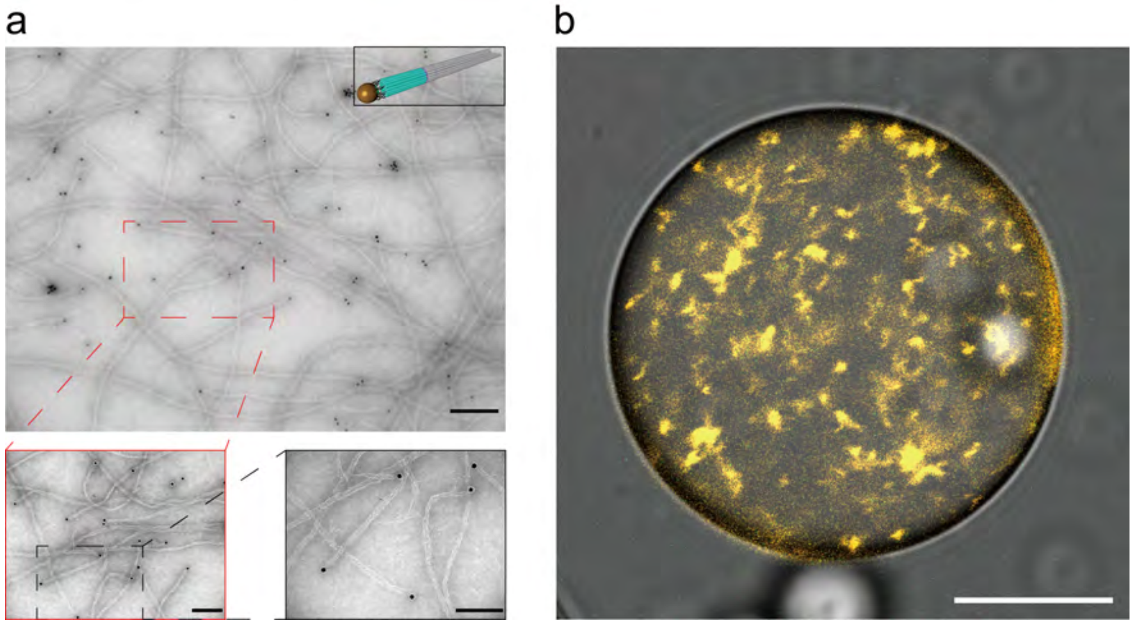

Figure 13: **a** TEM images demonstrating the successful implementation of the seeded growth mechanism as laid out in Fig. S12. The gold nanoparticles are clearly visible at one of the ends of the filaments, which extend from the DNA origami seeds. Scale bars: 200 nm (top image) and 100 nm (bottom images). **b** Confocal fluorescence microscopy image of the seeded DNA filaments encapsulated inside a water-in-oil droplet. Scale bar: 20 μm.

Supplementary Figure S14: Size distribution of the SUVs

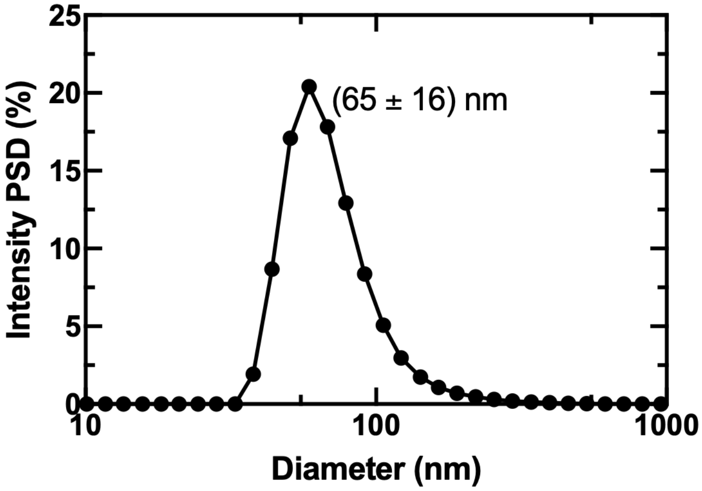

Figure 14: Intensity particle size distribution (PSD) of the SUV diameter determined by dynamic light scattering (DLS). The measurements have been performed using a solution of 1 mL of 200 µM SUVs. The average diameter and the standard deviation have been determined from 3 individual runs each containing 14 measurements. The distribution reveals a mean diameter of 65 ± 16 nm.

Supplementary Figure S15: STED imaging confirms the binding of the SUVs to the DNA-based filaments

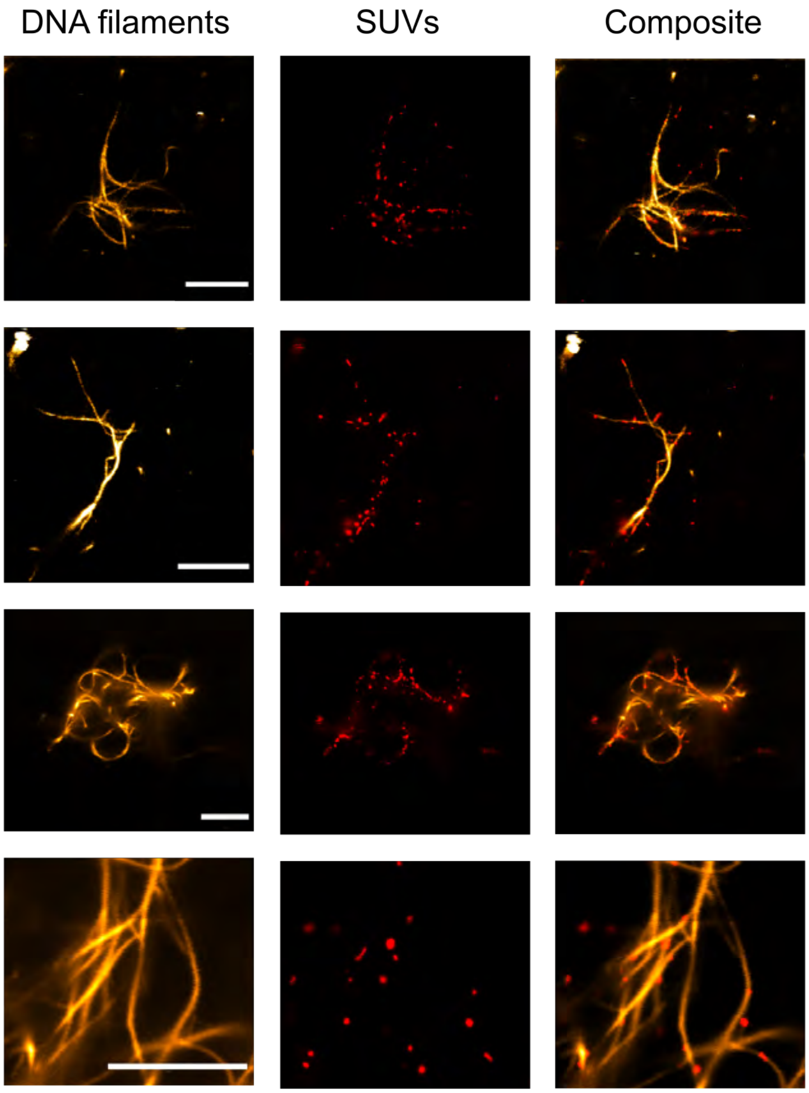

Figure 15: STED imaging confirms binding of the SUVs to the DNA filaments. STED images of the Cy3-labeled DNA filaments (λ_{ex} =560 nm), Atto633-labeled SUVs (λ_{ex} =640 nm) as well as composite images. SUVs colocalize with DNA filaments. Scale bars: 5 µm.

Supplementary Figure S16: Color-coded z-projections of the DNA-SUV networks

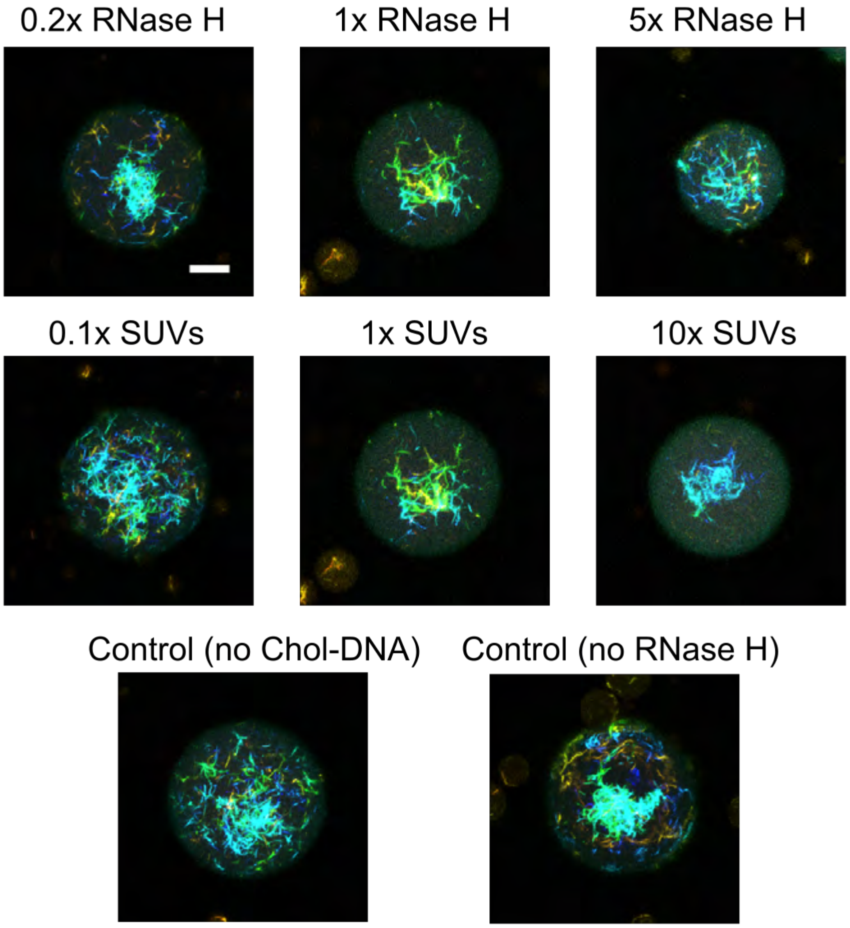

Figure 16: Color-coded confocal z-projections of the DNA networks (λ_{ex} =561 nm) in the presence of SUVs reconstructed from the confocal microscopy images at t = 0 min. From the images one can deduce the tendency of the DNA filaments to form more compact networks with a higher SUV density, possibly due to crosslinking of multiple DNA filaments by single SUVs. 1× RNase H corresponds to 5 units of RNase H and 1× SUVs corresponds to a concentration of 25 pM. Scale bar: 20 µm.

Supplementary Figure S17: Composite images of the DNA-SUV networks

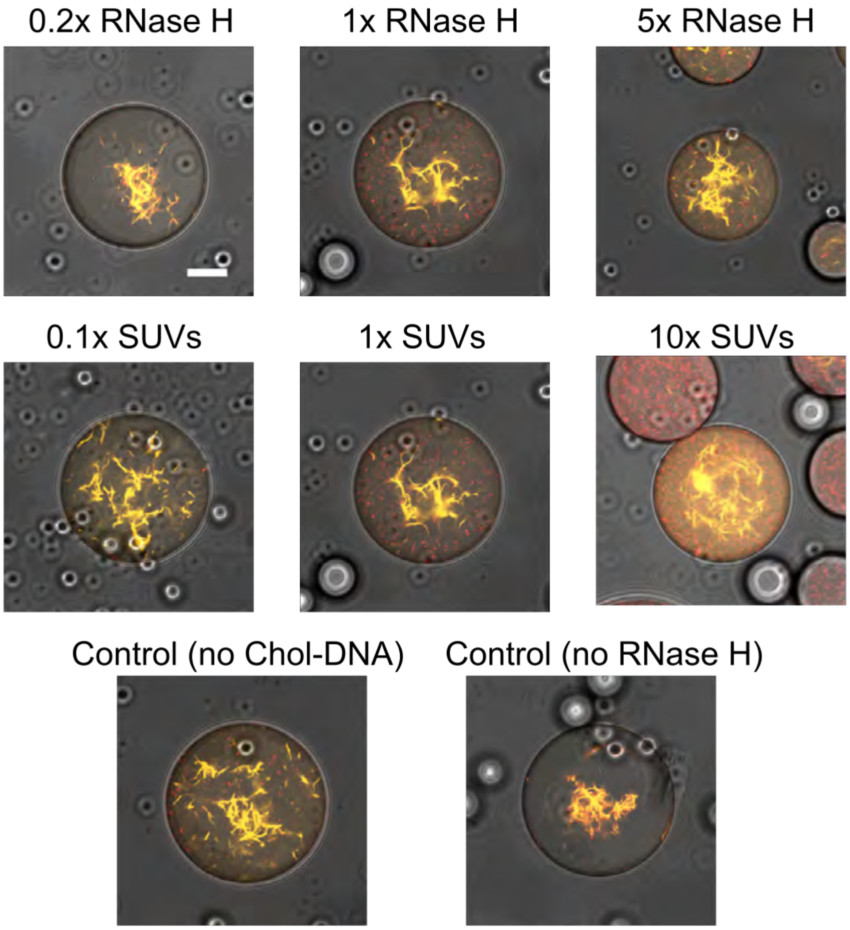

Figure 17: Composite bright-field and confocal microscopy images of the DNA-SUV networks (Cy3-labeled DNA, λ_{ex} =561 nm, orange or Atto633-labeled SUVs λ_{ex} =640 nm, red) encapsulated into water-in-oil droplets at t = 0 min. The images show the loss of fluorescence of the DNA filaments already at t = 0 min as well as complete binding of SUVs to the DNA filaments at low SUV concentrations. 1× RNase H corresponds to 5 units of RNase H and 1× SUVs corresponds to a concentration of 25 pM. Scale bar: 20 µm.

Supplementary Figure S18: DNA filaments remain intact after transport

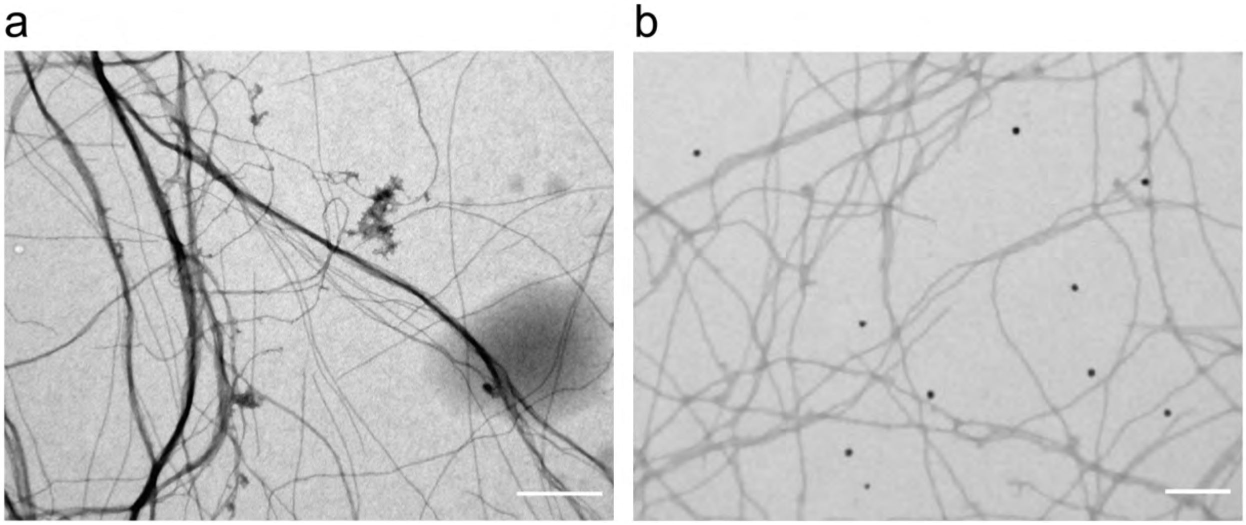

Figure 18: TEM images of the DNA-based filaments after apparent cargo transport of **a** SUVs and **b** gold nanoparticles. The filaments have been released from the droplets after cargo transport by breaking them up with addition of perfluoro-1-octanol (PFO) destabilizing agent.[7] The filaments remain intact after transport, confirming that the decrease in porosity $((1-\Phi)\cdot 100\%)$ is not due to filament disassembly. The fluorophores have been cleaved from the filaments, while leaving the filaments intact. Furthermore, the cargos (SUVs or gold nanoparticles) have detached from the filaments after cargo transport. Scale bar: 200 nm.

Supplementary Figure S19: Control experiments for SUV transport

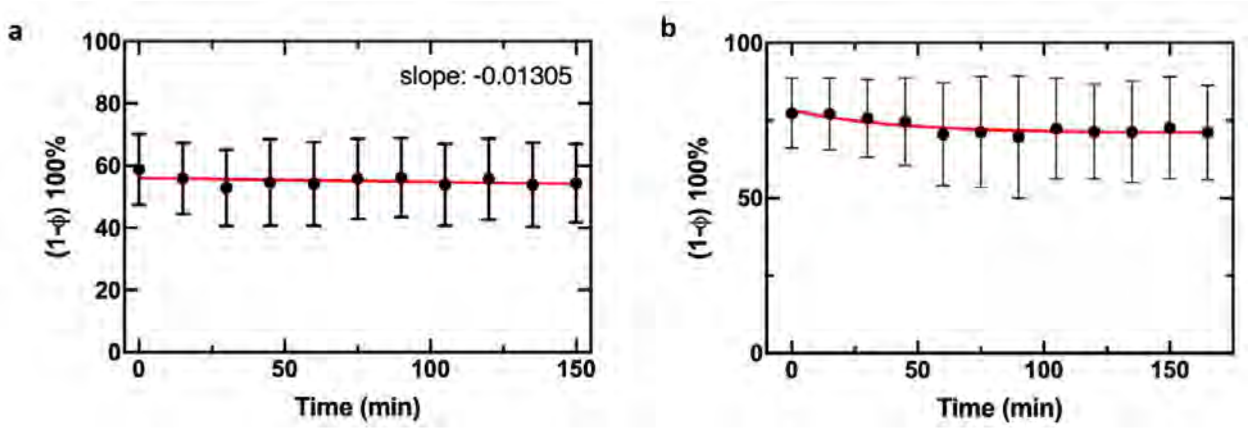

Figure 19: **a** Porosity ($(1-\Phi)\cdot 100\%$) in droplets over time without RNase H (mean +/- standard deviation for n = 4 droplets). The red line represents a linear fit, the decay corresponds to the rate of photobleaching. Photobleaching only causes a decrease of the porosity by 2 % over the course of 150 minutes. **b** Porosity ($(1-\Phi)\cdot 100\%$) in droplets over time without cholesterol-tagged DNA in the presence of RNase H (mean +/- standard deviation for n = 12 droplets). In this control experiment, the SUVs do not bind to the DNA filaments. Hence, transport should not occur and any reduction in $(1-\Phi)\cdot 100\%$ can be attributed to either photobleaching or unspecific cutting of the RNase H.

Supplementary Figure S20: TEM images of the gold nanoparticles attached to the DNA filaments

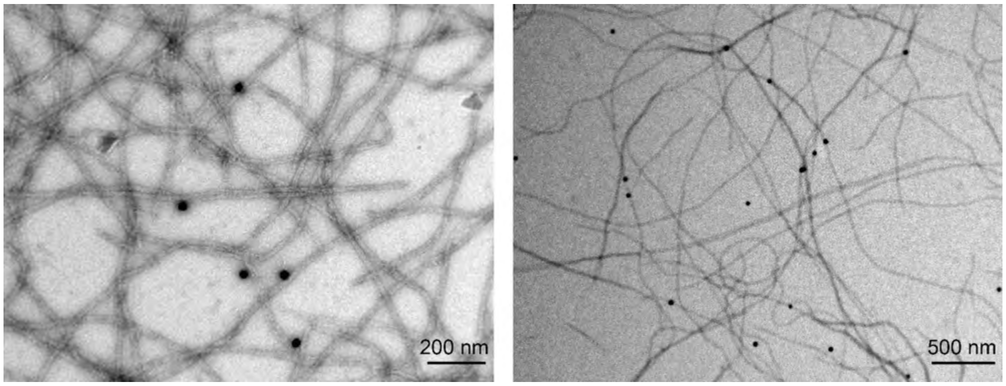

Figure 20: TEM images of the gold nanoparticles attached to the DNA filaments.

Supplementary Figure S21: TEM indicates rolling of gold nanoparticles along the DNA-based filaments

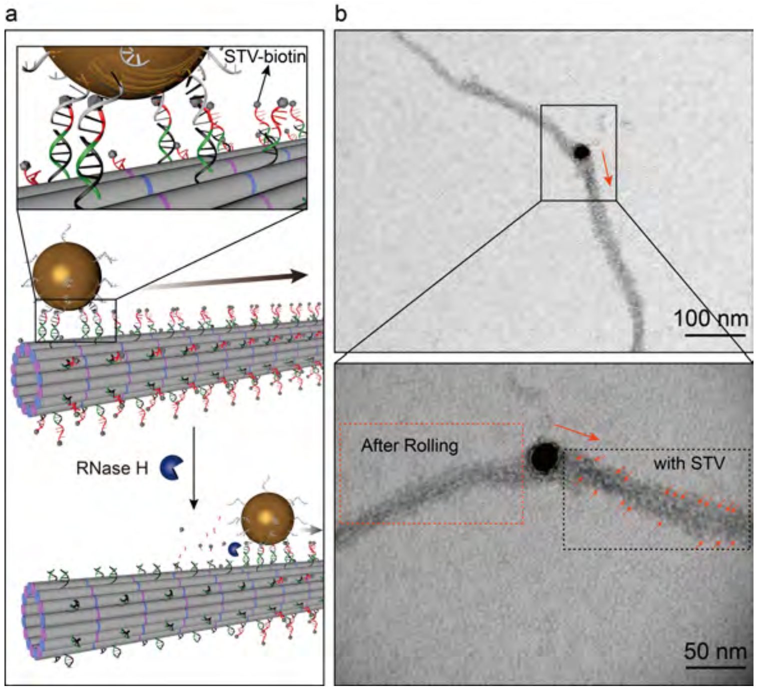

Figure 21: **a** Schematic of gold nanoparticles by rolling motion using RNase H mediated cleaving[4] of the RNA part of an RNA-DNA duplex on a DNA filament. The biotin functionalized single-stranded RNA-DNA (red-green, binds to streptavidin (STV)) is anchored on the filaments through DNA-DNA hybridization (black and green). The DNA strands (gray) on the gold nanoparticle hybridize with the RNA part. Upon addition of RNase H, the DNA-RNA double helix is cut, and STV is released. **b** TEM images of the filaments with STVs after adding RNase H. The dotted red frame demonstrates that RNA strands with STVs are cut since no STVs are attached on the filaments. The dotted black frame shows anchored STVs on the filaments without cutting.

Supplementary Figure S22: Mechanism of motion

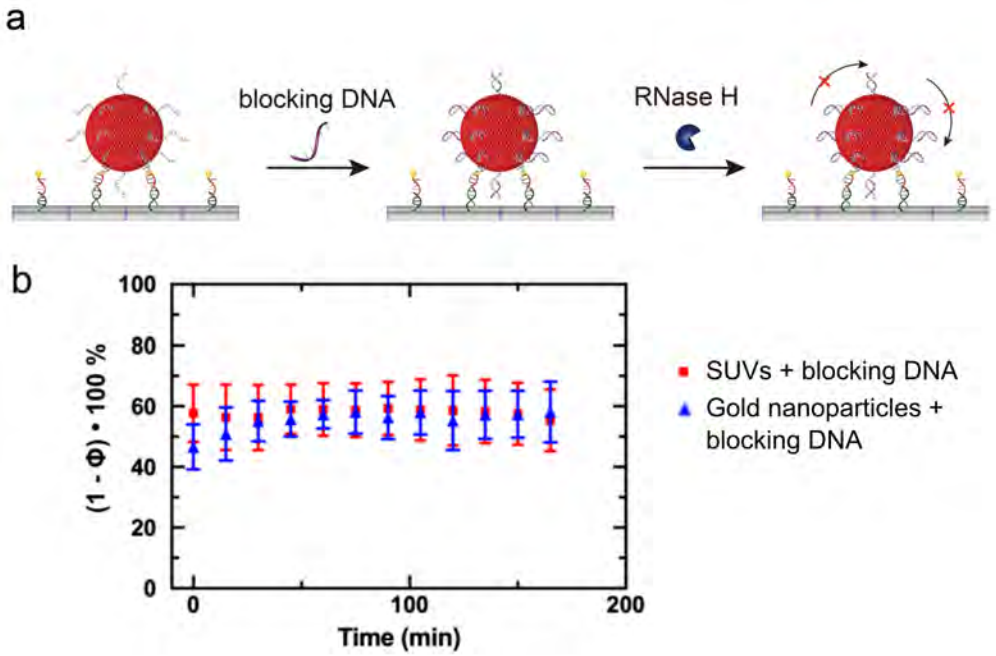

Figure 22: Mechanism of the particle motion. **a** Strategy to verifying how SUVs move along the DNA filaments. To demonstrate whether the SUVs roll, hop or glide along the DNA filaments, the free DNA on the SUVs is blocked by hybridization with blocking DNA strands (purple). The same experiment has been performed for gold nanoparticles. **b** Porosity ($(1-\Phi)\cdot 100\%$) measurements in the presence of the blocking DNA strands for gold nanoparticles (blue) and SUVs (red), respectively. The measured porosity remains nearly constant in each case. This indicates that the cargo transport along the filaments will halt, when the rolling motion is inhibited.

Supplementary Figure S23: DNA density on gold nanoparticles

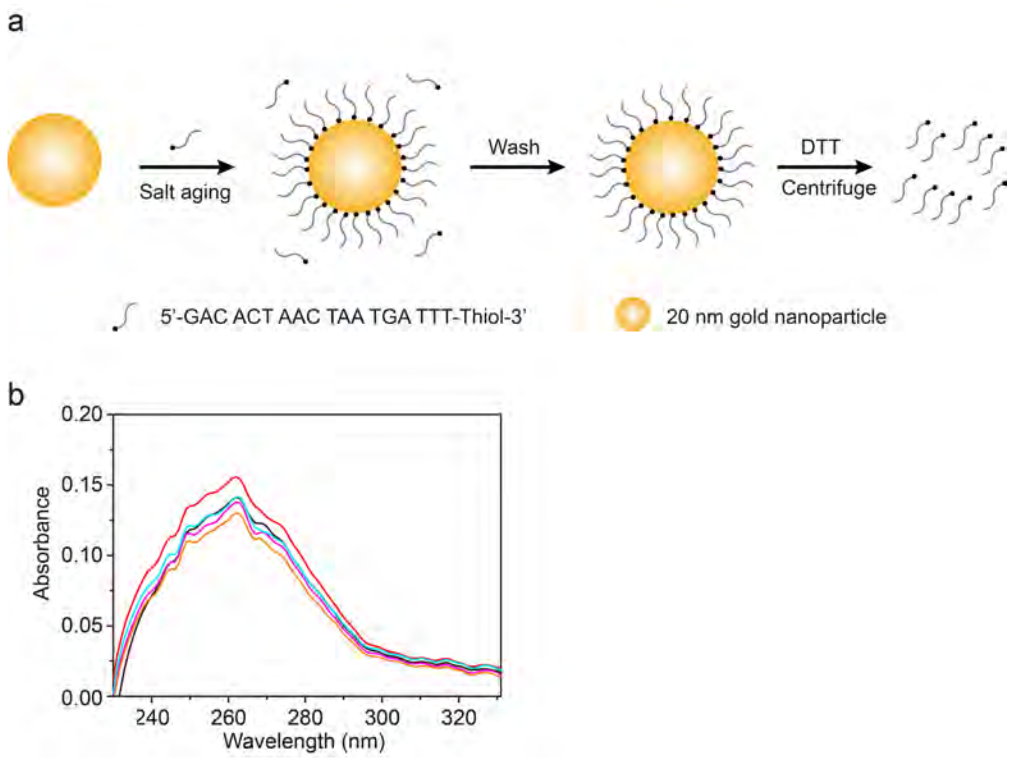

Figure 23: Quantification of the DNA density on gold nanoparticles. **a** Illustration of the experimental procedure used for the quantification of the DNA density on gold nanoparticles.[8] **b** UV-Vis spectra of the released DNA (mean and standard deviation from n=5 independent experiments). DNA coverage of 126±10 strands per gold nanoparticle or a density of 0.10±0.01 strands per nm^2 is obtained.

Supplementary Figure S24: DNA density on SUVs

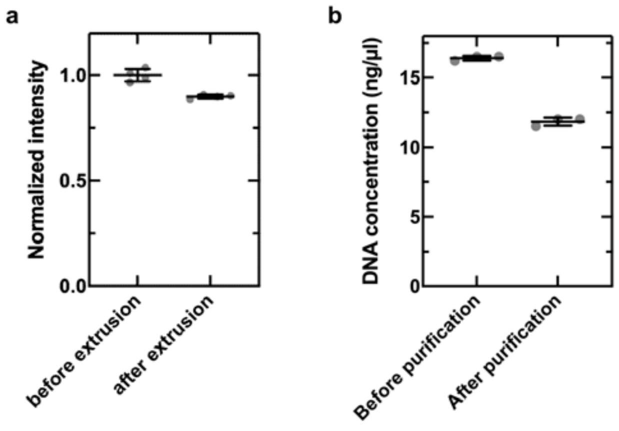

Figure 24: Quantification of the DNA density on SUVs. **a** Quantification of the loss of lipids after SUV extrusion. The fluorescence intensity of the lipid mixture (99% DOPC, 1% Atto633-DOPE) is determined before and after extrusion. 9.07% of the lipids are lost during the extrusion process (mean and standard deviation from n=4 independent measurements). **b** Incorporation efficiency of single-stranded cholesterol-tagged DNA into SUVs. The concentration of DNA is determined from UV absorbance measurements. The reference measurement is taken before addition to SUVs ("before purification"). Afterwards, the cholesterol-tagged DNA is incubated in excess with SUVs. The DNA concentration in the supernatant is measured ("after purification"). It corresponds to the unbound fraction of DNA (mean and standard deviation from n=3 independent experiments). 27.8±2.2% of 2 μM cholesterol-tagged DNA binds to SUVs (10 μM lipids before extrusion). This gives rise to a DNA density on the SUVs of 0.18±0.01 DNA strands per nm^2.

Supplementary Figure S25: Influence of the RNase H concentration on the SUV transport

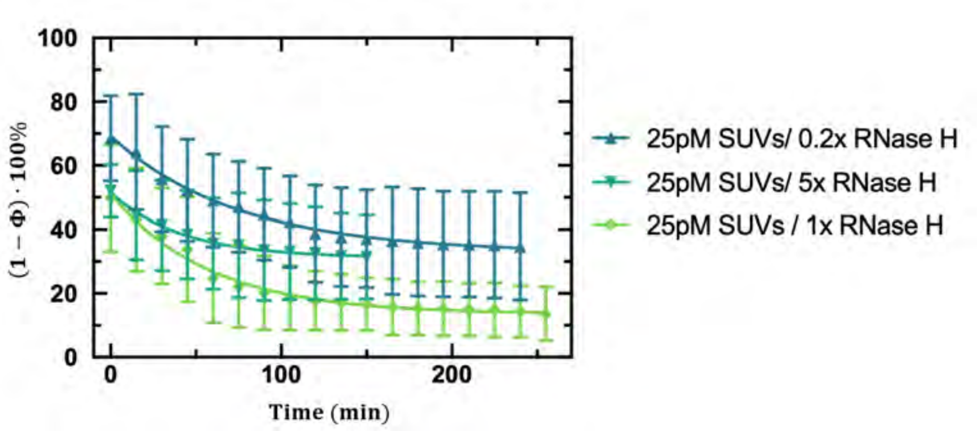

Figure 25: Influence of the RNase H concentration on the apparent SUV transport. Porosity ($(1 - \Phi) \cdot 100\%$, as a measure for the SUV transport) of the DNA filaments over time at different RNase H concentrations. 1× RNase H corresponds to 5 units of RNase H. Less RNase H (0.2×) or more RNase H (5×) lead to a decrease in transport. This could be due to the high viscosity of the RNase H solution, which contains 50 % glycerol and thus reduces diffusion speeds.[9]

Supplementary Figure S26: Decay constants for the DNA network fluorescence decrease

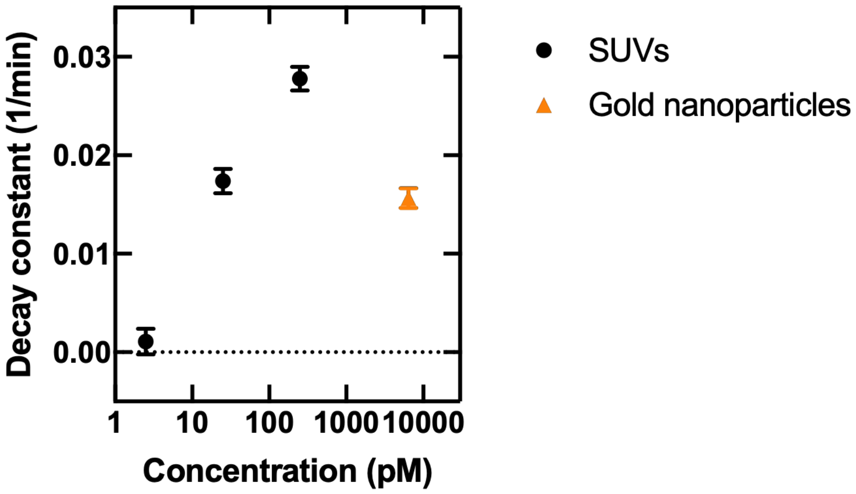

Figure 26: Decay constants for the DNA network fluorescence decrease. The porosity decrease over time is fitted with a one phase exponential decay revealing the apparent decay constant of the network fluorescence. The decay constant increases from negligible decay for 2.5 pM SUVs to 0.017 ± 0.001 1/min for 25 pM SUVs and to 0.028 ± 0.001 1/min for 250 pM SUVs. In the presence of 6 nM gold nanoparticles the decay constant is found to be 0.016 1/min.

Supplementary Videos

Supplementary Video S1: Dynamics of the toehold-modified DNA filaments inside water-in-oil droplets

Confocal timelapse of 500 nM Cy3-labelled toehold-modified DNA filaments ($\lambda_{ex} = 561$ nm) in a 1× TAE buffer containing 12 mM $MgCl_2$. After polymerization, the DNA filaments remain highly dynamic with constant rearrangement and remodelling. The video corresponds to the filaments shown in Fig. 2c. Scale bar: 50 µm.

Supplementary Video S2: Time-resolved polymerization of the ATP-aptamer-modified DNA filaments inside water-in-oil droplets

Confocal time series of 1 µM Cy3-labelled DNA filaments ($\lambda_{ex} = 561$ nm) in a 1× TAE buffer containing 20 mM $MgCl_2$ and 2 mM ATP. Over the course of 150 min, DNA filaments assemble inside the surfactant-stabilized water-in-oil droplets. The corresponding quantification of the porosity (i.e. the degree of polymerization) is shown in Fig. 3b (orange curve). Scale bar: 10 µm.

Supplementary Video S3: Time-resolved polymerization of actin filaments inside water-in-oil droplets

Confocal time series of actin-containing water-in-oil droplets (10 µM) labeled with phalloidin-rhodamine (λ_{ex} =561 nm). The video depicts a colour-coded z-projection of actin fluorescence over the course of 90 min. In the presence of ATP-containing actin polymerization buffer (see Methods), actin polymerizes over time into micron-sized filaments. The corresponding quantification of the porosity (i.e. the degree of polymerization) is shown in Fig. 3b (red curve). Scale bar: 20 µm.

Supplementary Video S4: Dynamics of the DNA filaments with aptamers for nucleolin and ATP targets inside water-in-oil droplets

Confocal time series of 500 nM Cy3-labelled DNA filaments ($\lambda_{ex} = 561$ nm) in a 1× TAE buffer containing 20 mM $MgCl_2$ and 1.5 µM nucleolin. Like the toehold-modified filaments (Video S1), the aptamer-functionalized DNA filaments remain highly dynamic with constant rearrangement and remodelling after polymerization. Scale bar: 20 µm.

Supplementary Video S5: SUV transport on DNA filaments inside water-in-oil droplets

Confocal time series of 250 nM Cy3-labeled DNA filaments (λ_{ex} = 561 nm, orange) and 25 pM Atto633-labeled SUVs (λ_{ex} = 633 nm, red) inside a water-in-oil droplet containing 0.25 units/µL of RNase H and 1× RNase H reaction buffer. The DNA filaments lose their fluorescence over time due to apparent RNase-mediated SUV transport. Scale bar: 20 µm.

References

(1) Green, L.; Amodio, A.; Subramanian, H. K. K.; Ricci, F.; Franco, E. pH-driven reversible self-assembly of micron-scale DNA scaffolds. *Nano Letters* **2017**, *17*, 7283–7288.

(2) Green, L. N.; Subramanian, H. K. K.; Mardanlou, V.; Kim, J.; Hariadi, R. F.; Franco, E. Autonomous dynamic control of DNA nanostructure self-assembly. *Nature Chemistry* **2019**, *11*, 510–520.

(3) Huizenga, D. E.; Szostak, J. W. A DNA Aptamer That Binds Adenosine and ATP. *Biochemistry* **1995**, *34*, 656–665.

(4) Yehl, K.; Mugler, A.; Vivek, S.; Liu, Y.; Zhang, Y.; Fan, M.; Weeks, E. R.; Salaita, K. High-speed DNA-based rolling motors powered by RNase H. *Nature Nanotechnology* **2015**, *11*, 184–190.

(5) Platzman, I.; Janiesch, J.-W.; Spatz, J. P. Synthesis of Nanostructured and Biofunctionalized Water-in-Oil Droplets as Tools for Homing T Cells. *Journal of the American Chemical Society* **2013**, *135*, 3339–3342.

(6) Mohammed, A. M.; Schulman, R. Directing self-assembly of DNA nanotubes using programmable seeds. *Nano Letters* **2013**, *13*, 4006–4013.

(7) Göpfrich, K.; Haller, B.; Staufer, O.; Dreher, Y.; Mersdorf, U.; Platzman, I.; Spatz, J. P. One-Pot Assembly of Complex Giant Unilamellar Vesicle-Based Synthetic Cells. *ACS Synthetic Biology* **2019**, *8*, 937–947.

(8) Baldock, B. L.; E, H. J. UV–Visible Spectroscopy-Based Quantification of Unlabeled DNA Bound to Gold Nanoparticles. *Anal. Chem.* **2016**, *88*, 12072–12080.

(9) D'Errico, G.; Ortona, O.; Capuano, F.; Vitagliano, V. Diffusion Coefficients for the Binary System Glycerol Water at 25 °C. A Velocity Correlation Study. *Journal of Chemical & Engineering Data* **2004**, *49*, 1665–1670.
